# Endocrine, Metabolic, and Skeletal Muscle Proteomic Responses During Energy Deficit With Concomitant Aerobic Exercise in Humans

**DOI:** 10.1096/fj.202502384RR

**Published:** 2025-11-03

**Authors:** Yusuke Nishimura, Carl Langan‐Evans, Harry L. Taylor, Wee L. Foo, James P. Morton, Sam Shepherd, Juliette A. Strauss, Jatin G. Burniston, José L. Areta

**Affiliations:** ^1^ Faculty of Health, Innovation, Technology and Science Tom Reilly Building, Liverpool John Moores University Liverpool UK

**Keywords:** aerobic exercise, dietary restriction, fibrosis, human evolution, lifespan, mitochondria, proteome, skeletal muscle, weight loss

## Abstract

Energy deficit is a potent physiological stressor that has shaped human evolution and can improve lifespan and healthspan in a wide range of species. Preserving locomotive capacity was likely essential for survival during the human hunter‐gatherer period but surprisingly little is known about the molecular effects of energy deficit on human skeletal muscle, which is a key tissue for locomotion and metabolic health. Here we show that after a 5‐day 78% reduction in energy availability with concomitant aerobic exercise in healthy men there was a profound modulation of skeletal muscle phenotype alongside increases in fat oxidation at rest and during exercise and a 2.1 ± 0.8 kg loss of fat free mass and 0.8 ± 0.6 kg of fat mass. We used stable isotope (D_2_O) labelling and peptide mass spectrometry to investigate the abundance and turnover rates of individual proteins. Abundance (1469 proteins) and synthesis rate (736 proteins) data discovered a shift toward a more oxidative phenotype and reorganization of cytoskeleton and extracellular matrix structure during energy deficit. Mitochondrial components: TCA, electron transport chain and beta‐oxidation, were prominently represented amongst proteins that increased in abundance and synthesis rate, as well as proteins related to mitochondrial proteostasis, remodeling and quality‐control such as BDH1 and LONP1. Changes in muscle metabolic pathways occurred alongside a reduction in extracellular matrix proteins, which may counteract the age‐related muscle fibrosis. Our results suggest that muscle metabolic pathways are not only preserved but positively affected during periods of concomitant low energy availability and exercise.

## Graphical abstract

After consecutive 5‐day periods of energy balance and energy deficit (ED, 78% reduced energy availability—EA) including aerobic exercise, trained males experienced reduced fat and fat‐free mass, alongside hormonal and metabolic adaptations favoring energy preservation and greater fat oxidation at rest and during exercise, without changes in resting metabolic rate. Skeletal muscle proteomics revealed unchanged sarcoplasmic and myofibrillar protein synthesis rates but increased mitochondrial proteins. Protein abundance data confirmed higher mitochondrial content and complex modulation of extracellular matrix, indicating metabolic and structural adaptations. Figure created in BioRender (https://BioRender.com/2lvzxwi).

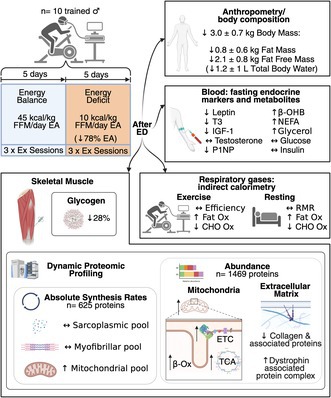

## 1 Introduction

During human evolution hominids may have adapted to endure periods of intermittent food availability alongside sustained needs for high levels of physical activity to procure food, shelter, or evade danger [1, 2]. In the face of limited energy availability, life history models of energy allocation predict that energetic resources will be re‐distributed amongst competing physiological processes in an order of priority that maximizes survival [3]. In the ancestorial human habitat (that of hunter‐gatherers), suppression of growth and reproductive function would spare limited energetic resources during periods of food scarcity [4], whereas a reduction in physical capacity (necessary for food procurement) would have been less favorable [5]. Given also the remarkable capacity for endurance in the genus Homo, possibly in relation to persistence hunting and scavenging [6, 7], human physiology may have adapted to prioritize endurance capacity. Thus, the physiological machinery that supports locomotion may be preserved even during low energy availability, at the expense of other energetically demanding processes such as growth and reproductive function.

Energy prioritization has far‐reaching implications for several modern populations where energy deficit is either intentional or unavoidable. In healthy aging, energy restriction is explored for its potential to extend lifespan and healthspan [8, 9]; in athletic populations, low energy availability is linked to adverse effects on health and performance [10, 11, 12]; in overweight or obese individuals, energy deficit underpins interventions aimed at improving metabolic health through weight loss [13]; and in space exploration, astronauts are reported to be in energy deficit, putting long‐duration missions at risk [14]. A common and critical tissue in all these scenarios is skeletal muscle—essential for locomotion and central to metabolic regulation.

The crucial role of skeletal muscle during energy deficit has recently come to the forefront of public attention due to the growing use of GLP‐1 receptor agonists, which are effective for weight loss, but can cause substantial muscle loss [15]. It is not clear, however, whether muscle loss associated with GLP‐1 receptor agonists is clinically relevant [16]. A comprehensive analysis of how energy deficit affects skeletal muscle phenotype alongside the endocrine and physiological responses of energy preservation is yet to be conducted. An enhanced mechanistic understanding of the skeletal muscle phenotype shift with energy restriction and concomitant exercise in humans will not only identify key pathways modulated by energy deficit but also help to understand and predict the potential risks and benefits of interventions aiming to enhance muscle quality and function in the face of energy deficit.

Earlier studies that have investigated the effect of energy deficit on skeletal muscle proteins in humans are limited to skeletal muscle bulk protein synthesis/breakdown [17, 18, 19, 20, 21, 22], provide limited insights through addressing selected molecular markers [23, 24, 25], provided a comprehensive molecular response but only the gene expression level [8, 26, 27], or investigated protein turnover of individual proteins in energy deficit compared only to a testosterone treatment group [28]. To the best of our knowledge, research reporting the effects of energy deficit in skeletal muscle proteome turnover and abundance against energy balance has not yet been conducted in humans. Protein turnover is a highly responsive characteristic of the muscle proteome that is required to maintain protein homeostasis and underpins the process of muscle adaptation. The processes of protein turnover (e.g., ribosomal translation, protein chaperoning, and degradation via the ubiquitin proteasome system) are energetically costly and highly responsive to changes in muscle activity and feeding. Muscle proteins exhibit a wide range of different turnover rates and no study to date has investigated skeletal muscle proteome dynamics comprehensively in response to energy deficit. Dynamic proteomic profiling is a new technique built on the methodological developments in the fields of stable isotopic labelling, proteomics and computational biology, allowing to determine the synthesis, abundance and degradation of human skeletal muscle proteins on a protein‐by‐protein basis [29, 30]. Dynamic proteome profiling can characterize hundreds of skeletal muscle proteins to systematically and agnostically study tissue‐specific protein dynamics [31, 32], which may help determine the effect of concomitant energy deficit and exercise on skeletal muscle protein turnover and abundance.

In this study, we provide an integrated physiological, endocrine, and metabolic assessment in parallel with skeletal muscle dynamic proteome profiling in response to a 5‐day pronounced energy deficit intervention with concomitant aerobic‐type exercise, compared to an energy balance intervention in humans. Our analysis evidenced key physiological, endocrine, and metabolic responses coherent with an energy preservation response triggered by energy deficit and increase in fatty acid oxidation, with skeletal muscle showing two distinct responses: a clear upregulation of mitochondrial proteins synthesis and abundance, and a downregulation of extracellular matrix and cytoskeletal proteins. Our findings corroborate and expand animal and human findings suggesting that energy deficit improves skeletal muscle quality and leads to a more youthful phenotype through enhancement of mitochondrial proteome quality and reduction of extracellular matrix proteins associated to age‐related fibrosis [23, 33, 34, 35].

These findings provide mechanistic support for the use of energy restrictive diets with concomitant exercise for the enhancement of skeletal muscle phenotype and yield insights into metabolic pathways with the potential to derive tailored therapeutic support to mimic the effects of energy deficit in skeletal muscle.

## 2 Methods

### 2.1 Participants

**FIGURE 1:**
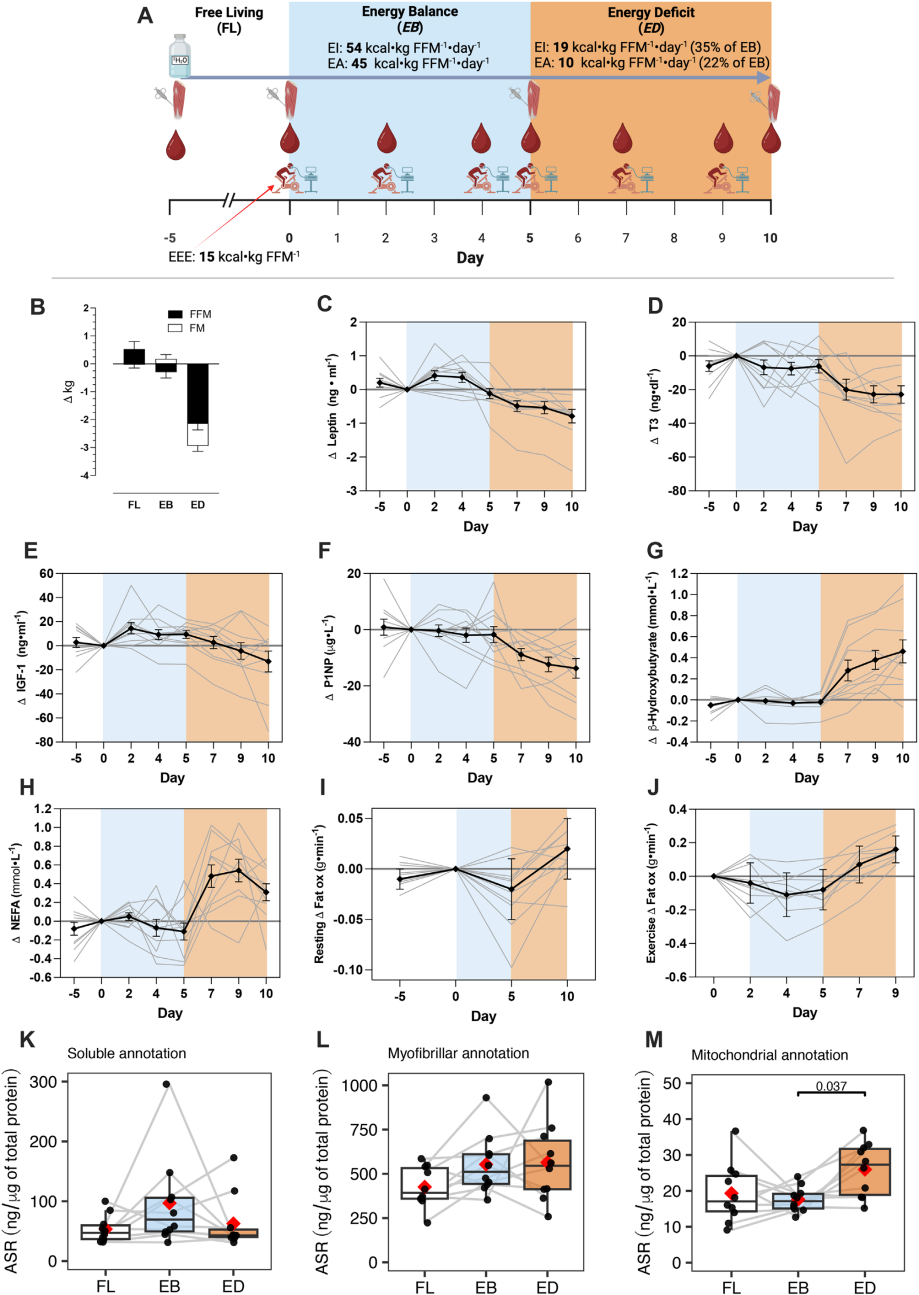
Study design and key metabolic variables summary. (A) Schematic overview of the study design, created with http://biorender.com. (B) Change (mean ± SEM) of body fat‐free mass (FFM) and fat mass (FM) between the start and end of each 5‐day period. (C–J) Change (mean ± SEM) relative to Day 0 (beginning of controlled intervention phase) in (C) plasma leptin, (D) serum triiodothyronine (*T*_*3*_), (E) serum insulin‐like growth factor 1 (*IGF‐1*), (F) plasma procollagen type 1 N‐terminal propeptide (*P1NP*), (G) plasma β‐Hydroxybutyrate, (H) plasma non‐esterified fatty acid (*NEFA*) concentrations, (I) resting fat oxidation, and (J) mean fat oxidation rates during steady state exercise at ~60% V̇O_2max_ of ~90 min exercise. Absolute values and statistical analyses are presented in Table 2. *n* = 10 (individuals) × 4, 6, or 8 (time‐points). Details of resting and exercise variables are presented in Tables 2 and 3, respectively. (K–M) Absolute synthetic rate (*ASR*) of proteins annotated into (K) soluble, (L) myofibrillar, and (M) mitochondrial assessed *in silico*. The box plot represents the interquartile range (IQR; 25th–75th percentile), with the horizontal line indicating the median. Whiskers extend to the minimum and maximum values within 1.5× IQR. ASR (ng/μg of protein) values for individual subjects are shown as black data points. Gray lines connect values from the same subject across the three periods (FL, EB, and ED). The red diamond represents the mean. Pairwise comparisons of the estimated marginal means for the experimental period were conducted using the Bonferroni adjustment for multiple comparisons in the linear mixed‐effects model. EA, energy availability; EI, energy intake; EEE, exercise energy expenditure.

Ten healthy males that were active (completing > 3 sessions/h of aerobic exercise per week), weight‐stable (reported ≤ 2 kg fluctuation, over the previous 6 months) without known health complications were recruited for the study (see Table 1) after being pre‐screened and providing a written consent. Ethical approval for the study was granted by the NHS North‐West‐Liverpool Central Research Ethics Committee (Ref: 21/NW/0205). Participant characteristics are presented in Table 1, and Figure 1A provides a schematic of the experiment design (further detailed in Appendix S1 (Protocol)).

**TABLE 1:** Participants characteristics.

*N*—Males	10
Age [years]	25 (5)
Height [m]	1.80 (0.06)
Body mass [kg]	78.8 (8.2)
Body fat—DXA—[%]	18 (3.4)
V̇O_2_max [mL/kg/min]	52 (7.7)
Cycling aerobic peak power [W]	304 (62)

*Note:* Characteristics are given as mean (standard deviation).

### 2.2 Study Design

After participant characterization testing, participants followed a quasi‐experimental design intervention (Figure 1A) during a period of 15 consecutive days, which consisted of three consecutive 5‐day periods of, (1) free living (FL, Days −5 to −1), followed by two phases of controlled diet and exercise (2) energy balance (EB: Days 0–5), which aimed at maintaining body mass, and (3) energy deficit (ED: Days 5–10), which induced weight‐loss through a dietary energy deficit (Table S1). We sampled fasting blood, resting metabolic rate, and respiratory gases during morning exercise, and skeletal muscle (vastus lateralis of quadriceps) before and after each period (Figure 1A; and Appendix S1 (Protocol)).

### 2.3 Participant Characterization Assessment

Prior to the start of the study, participants reported to the laboratory for assessment of anthropometric parameters, cycloergometer‐based (Lode Corival cpet: Lode, Groningen, Netherlands) maximal oxygen consumption (V̇O_2Max_; Moxus Modular Metabolic System, AEI Technologies, Pittsburgh, PA, USA), peak power output (PPO), lactate threshold (LT; Biosen C‐Line, EKF Diagnostic, Cardiff, UK), and submaximal O_2_ utilization using standard protocols to determine participant fitness and the workload for exercise intensity during intervention [36].

### 2.4 Dietary and Exercise Intervention Phase With Manipulation of EA (Days 0–10)

From Day 0 to Day 10, all food was provided to participants in custom‐made, prepackaged meals, and participants consumed all and only the foodstuff provided, with non‐calorie containing drinks allowed ad libitum. This period consisted of two 5‐day phases; Days 0–4 (inclusive) comprised the energy balance (EB) dietary provision, with Days 5–9 (inclusive), of energy deficit (ED) diet containing 35% of the energy intake of EB. The meals of each intervention phase started after the morning (fasted) measurements and exercise session of Days 0 and 5, therefore measurements of Days 0, 5, and 10 correspond to FL, EB, and ED diets respectively. Proportion of macronutrients was clamped at 60% carbohydrate, 20% fat, and 20% protein in both diets (see diet details in Table S1). During each phase, individuals completed three cycling sessions to expend 15 kcal·kg fat‐free mass^−1^·day^−1^. Average daily energy availability ((Energy intake—Exercise energy expenditure)·kg FFM^−1^) [37] achieved was 45 and 10 kcal·kg FFM^−1^·day^−1^ for the EB & ED phases, respectively, which represents a 78% reduction in energy availability in ED.

### 2.5 Study Intervention Phase (Days −5 to 10): Assessments and Biological Samples Collection

For all testing sessions (Days −5, 0, 2, 4, 5, 7, 9, and 10), participants arrived at the laboratory at 0700–0800 h in a fasted state. Resting metabolic rate (RMR) was assessed using indirect calorimetry (GEM Open Circuit Indirect Calorimeter; GEMNutrition Ltd., Warrington, UK) following a standard 25‐min protocol [38]. Body composition was assessed via bio‐electrical impedance analysis (*BIA*; SECA mBCA 515: SECA GMBH, Hamburg, Germany), on each laboratory visit, and via a whole‐body dual‐energy X‐ray absorptiometry scan (*DXA*; Hologic, Manchester, United Kingdom) on Days −5, 0, 5, and 10 using standardized procedures [39]. Fasting venous blood samples were collected at each laboratory visit, and skeletal muscle was obtained from the lateral portion of the vastus lateralis of the quadriceps muscle after each RMR assessment, and prior to exercise.

#### 2.5.1 Exercise Protocol

Exercise sessions were initiated with consecutive 3‐min stages at 50 W, 75 W, 100 W, and 125 W. Thereafter workload was set to a steady‐state power output to elicit 60% V̇O_2Max_ until achieving a target EEE of 15 kcal·kg FFM^−1^·day^−1^. Respiratory gas assessments were repeated every 15 min with rate of perceived exertion (RPE [40]), assessed at the end of each stage. Fat and carbohydrate oxidation were estimated based on the equations standard equations [41]. Participants were provided with a carbohydrate‐rich meal (30 g carbohydrates, < 1 g fat, and 3 g protein) at ~5–15 min before exercise and at 5 min breaks after 30 and 60 min of exercise.

### 2.6 Blood Analyses

Analysis of blood metabolites, hormones and bone turnover markers: Plasma glucose, lactate, non‐esterified fatty acids (NEFA), and glycerol concentrations were analyzed using commercially available kits and a Randox Daytona spectrophotometer (Randox, Crumlin, Ireland). Commercially available enzyme‐linked immunosorbent assays (ELISA) were used to measure serum total triiodothyronine (T3; DiaMetra S.r.l, Perugia, Italy), serum insulin‐like growth factor 1 (IGF‐1), plasma insulin, and plasma leptin (all DRG International Inc., Springfield, NJ, USA), serum erythropoietin (EPO; Abcam, Cambridge, UK) and serum growth/differentiation factor‐15 (GDF‐15; R&D Systems, Minneapolis, MN, USA). Bone turnover markers (plasma ß‐CTX and P1NP), and serum total testosterone concentrations were analyzed by a commercial laboratory (Liverpool Clinical Laboratories, Liverpool, UK).

### 2.7 Skeletal Muscle Proteomic Analysis

#### 2.7.1 Stable Isotope Labelling In Vivo

Biosynthetic labelling of newly synthesized proteins was achieved by oral consumption of deuterium oxide (D_2_O; Sigma‐Aldrich, UK). A “loading” + “maintenance” design was used to rapidly raise the enrichment of D_2_O in the participants body water compartment and then sustain a steady‐state level of enrichment throughout the experimental period. After collection of the baseline muscle sample on experimental Day −5, participants consumed doses of 200 mL, 150 mL, and 150 mL of 99.8 atom % of D_2_O at approximately 3‐h intervals across the day. On subsequent days, participants were provided with 50 mL “maintenance doses” of 99.8 atom % of D_2_O in sterile feeding bottles and were prompted to consume one dose each day by members of the research team.

#### 2.7.2 Calculation of D_2_O Enrichment

Body water enrichment of D_2_O was measured in plasma samples against external standards that were constructed by adding D_2_O to 18 MΩ water over the range from 0.0% to 5.0% in 0.5% increments. D_2_O enrichment of aqueous solutions was determined by gas chromatography–mass spectrometry after exchange with acetone [42]. Samples were centrifuged at 12,000 *g*, 4°C for 5 min, and 20 μL of plasma supernatant or standard was reacted overnight at room temperature with 2 μL of 10 M NaOH and 4 μL of 5% (v/v) acetone in acetonitrile. Acetone was then extracted into 500 μL chloroform and water was captured in 0.5 g Na_2_SO_4_ before transferring a 200 μL aliquot of chloroform to an auto‐sampler vial. Samples and standards were analyzed in triplicate by using an Agilent 5973 N mass selective detector coupled to an Agilent 6890 gas chromatography system (Agilent Technologies, Santa Clara, CA, USA). A CD624‐GC column (30 m 30.25 mm 31.40 mm) was used in all analyses. Samples (1 μL) were injected by using an Agilent 7683 auto sampler. The temperature program began at 50°C and increased by 30°C/min to 150°C and was held for 1 min. The split ratio was 50:1 with a helium flow of 1.5 mL/min. Acetone eluted at 3 min. The mass spectrometer was operated in the electron impact mode (70 eV) and selective ion monitoring of m/z 58 and 59 was performed by using a dwell time of 10 ms/ion.

#### 2.7.3 Protein Extraction and Quantification

Proteins were extracted from muscle samples as previously described [30, 31]. Muscle samples were ground in liquid nitrogen, then homogenized on ice in 10 volumes of 1% Triton X‐100, 50 mM Tris, pH 7.4 (including complete protease inhibitor; Roche Diagnostics, Lewes, United Kingdom) using a PolyTron homogenizer (KINEMATICA, PT 1200 E) followed by sonication. Homogenates were incubated on ice for 15 min, then centrifuged at 1000 × *g*, 4°C, for 5 min to fractionate myofibrillar (pellet) from soluble (supernatant) proteins. Myofibrillar proteins were resuspended in a half‐volume of homogenization buffer followed by centrifuged at 1000 × *g*, 4°C, for 5 min. The washed myofibrillar pellet was then solubilized in lysis buffer (7 M urea, 2 M thiourea, 4% CHAPS, 30 mM Tris, pH 8.5). Aliquots of protein were precipitated in 5 volumes of ice‐cold acetone and incubated for 1 h at −20°C, and resuspended in 200 μL of lysis buffer. Proteins were cleared by centrifugation at 12 000 × *g*, 4°C, for 45 min. Total protein concentration (μg/μL) was quantified against bovine serum albumin (BSA) standards using the Bradford assay (Thermo Scientific, #23236), according to the manufacturer's instructions.

#### 2.7.4 Protein Digestion

Tryptic digestion was performed using the filter‐aided sample preparation (FASP) method [43]. Aliquots containing 100 μg protein were washed with 200 μL of UA buffer (8 M urea, 100 mM Tris, pH 8.5). Proteins were incubated at 37°C for 15 min in UA buffer containing 100 mM dithiothreitol followed by incubation (20 min at 4°C) protected from light in UA buffer containing 50 mM iodoacetamide. UA buffer was exchanged for 50 mM ammonium bicarbonate and sequencing‐grade trypsin (Promega, Madison, WI, USA) was added at an enzyme to protein ratio of 1:50. Digestion was allowed to proceed at 37°C overnight then peptides were collected in 100 μL of 50 mM ammonium bicarbonate containing 0.2% (v/v) trifluoroacetic acid. Samples containing 4 μg of peptides were de‐salted using C18 Zip‐tips (Millipore Billercia, MA, USA) and eluted in 40% (v/v) acetonitrile and 0.1% (v/v) trifluoroacetic acid. Peptides were dried by vacuum centrifugation and resuspended in 20 μL of 2.5% (v/v) acetonitrile, 0.1% (v/v) formic acid containing 10 fmol/μL yeast alcohol dehydrogenase (MassPrep standard; Waters Corp., Milford, MA).

#### 2.7.5 Liquid Chromatography‐Mass Spectrometry Analysis

Peptide mixtures were analyzed using an Ultimate 3000 RSLC nano liquid chromatography system (Thermo Scientific) coupled to Q‐Exactive orbitrap mass spectrometer (Thermo Scientific). Samples were loaded on to the trapping column (Thermo Scientific, PepMapTM 100, 5 μm C18, 300 μm X 5 mm), using ulPickUp injection, for 1 min at a flow rate of 25 μL/min with 0.1% (v/v) TFA and 2% (v/v) ACN. Samples were resolved on a 500 mm analytical column (Easy‐Spray C18 75 μm, 2 μm column) using a gradient of 97.5% A (0.1% formic acid) 2.5% B (79.9% ACN, 20% water, and 0.1% formic acid) to 50% A 50% B over 150 min at a flow rate of 300 nL/min. The data‐dependent selection of the top‐10 precursors selected from a mass range of m/z 300–1600 was used for data acquisition consisted of a 70 000‐resolution full‐scan MS scan at m/z 200 (AGC set to 3e6 ions with a maximum fill time of 240 ms). MS/MS data were acquired using quadrupole ion selection with a 3.0 m/z window, HCD fragmentation with a normalized collision energy of 30 and in the orbitrap analyzer at 17 500‐resolution at m/z 200 (AGC target 5e4 ion with a maximum fill time of 80 ms). To avoid repeated selection of peptides for MS/MS, the program used a 30 s dynamic exclusion window.

#### 2.7.6 Label‐Free Quantitation of Protein Abundances

Progenesis Quantitative Informatics for Proteomics (QI‐P; Nonlinear Dynamics, Waters Corp., Newcastle, UK, Version 4.2) was used for label‐free quantitation, consistent with previous studies. Log‐transformed MS data were normalized by inter‐sample abundance ratio, and relative protein abundances were calculated using nonconflicting peptides only. In addition, abundance data were normalized to the three most abundant peptides of yeast ADH1 to obtain abundance estimates in fmol/μg total protein [44]. MS/MS spectra were exported in Mascot generic format and searched against the Swiss‐Prot database (2021_03) restricted to Homo‐sapiens (20 371 sequences) using locally implemented Mascot server (v.2.2.03; www.matrixscience.com). The enzyme specificity was trypsin with two allowed missed cleavages, carbamidomethylation of cysteine (fixed modification) and oxidation of methionine (variable modification). M/Z error tolerances of 10 ppm for peptide ions and 20 ppm for fragment ion spectra were used. Peptide results were filtered to 1% FDR based on decoy search and at least one unique peptide was required to identify each protein. The Mascot output (xml format), restricted to non‐homologous protein identifications was recombined with MS profile data in Progenesis.

### 2.8 Parallel Reaction Monitoring

For targeted analysis of PLIN2 and PLIN5 in human skeletal muscle, the instrument was operated in parallel reaction monitoring (PRM) mode using an Ultimate 3000 RSLCTM nano system (Thermo Scientific) coupled to a Q‐Exactive Orbitrap mass spectrometer (Thermo Scientific). Stable isotopically‐labeled (SIL) peptides corresponding to the targeted endogenous peptides were spiked in each sample as a 1 fmol/μL concentration and 0.5 fmol/μL for PLIN2 and PLIN5, respectively (HeavyPeptideTM AQUA Basic). Samples (4.5 μL corresponding to 1.8 ng of protein) were loaded on to the trapping column (Thermo Scientific, PepMap100, 5 μm C18, 300 μm X 5 mm), using μlPickUp injection, for 1 min at a flow rate of 100 μL/min with 2% (v/v) acetonitrile and 0.1% (v/v) formic acid. Samples were resolved on a 500 mm analytical column (Easy‐Spray C18 75 μm, 2 μm column) using a gradient of 97.5% A (0.1% formic acid) 2.5% B (79.9% ACN, 20% water, 0.1% formic acid) to 55% A 45% B over 31 min at a flow rate of 300 nL/min. Untargeted MS/MS analysis of peptides were acquired via HCD fragmentation mode (normalized collision energy 27) and detection in the Orbitrap (70 000 resolution at m/z 200, 5e4 automatic gain control (AGC), 240 ms maximum injection time, and 2 m/z isolation window) and a 30 s dynamic exclusion window was used to avoid repeated selection of peptides for MS/MS analysis.

Data were processed using Skyline‐daily v23.1.1.353. Product ion extraction ion chromatograms (XICs) were generated by including all matching scans for MS2 filtering. Ion match tolerance was set to 0.055 m/z and matched to 1+ for MS2 filtering of b‐ or y‐type ions that were reliably quantitative in all samples. All data were manually confirmed for co‐elution of MS1 and MS2 and ratio dot product (rdotp) ≥ 0.91 in all samples. Quantification was performed with MS2 fragments only and absolute abundance (fmol) was estimated by comparing the ratio of the signal intensities between the light peptide (endogenous) and heavy SIL peptide (L/H ratio) using a one‐point calibration.

### 2.9 Measurement of Protein Turnover Rates

Mass isotopomer abundance data were extracted from MS spectra using Progenesis Quantitative Informatics (Non‐Linear Dynamics, Newcastle, UK). Consistent with previous work [30, 31, 32], the abundances of peptide mass isotopomers were collected over the entire chromatographic peak for each proteotypic peptide that was used for label‐free quantitation of protein abundances. Mass isotopomer information was processed using in‐house scripts written in Python (version 3.12.4). The incorporation of deuterium into newly synthesized protein was assessed by measuring the increase in the relative isotopomer abundance (RIA) of the m_1_ mass isotopomer relative to the sum of the m_0_ and m_1_ mass isotopomers (Equation 1) that exhibits rise‐to‐plateau kinetics of an exponential regression [45] as a consequence of biosynthetic labelling of proteins in vivo.

(1) $$\mathrm{RIA}=\frac{m_{1}}{m_{0}+m_{1}}$$

The plateau in RIA (RIA_plateau_) of each peptide was derived (Equation 2) from the total number (*N*) of ^2^H exchangeable H—C bonds in each peptide, which was referenced from standard tables [46] and the difference in the D:H ratio (^2^H/^1^H) between the natural environment (DH_nat_) and the experimental environment (DH_exp_) based on the molar percent enrichment of deuterium in the precursor pool, according to [47].

(2) $${\mathrm{RIA}}_{\text{plateau}}=1-\left(\frac{1}{\left(\frac{1}{1-\mathrm{RIA}\left(t_{0}\right)}\right)+N\left({\mathrm{DH}}_{\exp}-{\mathrm{DH}}_{\mathrm{nat}}\right)}\right)$$

The rate constant of protein degradation (*k*_deg_) was calculated (Equation 3) between the beginning (t_0_) and end (t_1_) of each 5‐day labelling period. Calculations for exponential regression (rise‐to‐plateau) kinetics reported in [47] were used and *K*_deg_ data were adjusted for differences in protein abundance (*P*) between the beginning (*t*_0_) and end (*t*_1_) of each labelling period.

(3) $$k_{\deg}=-\frac{1}{t-t_{0}}•\ln\left(1-\frac{\mathrm{RIA}\left(t_{1}\right)-\mathrm{RIA}\left(t_{0}\right)}{\;{\mathrm{RIA}}_{\text{plateau}}-\mathrm{RIA}\left(t_{0}\right)}\;\right)⦁\frac{\;P\left(t\right)}{P\left(t_{0}\right)}$$

The absolute abundance (*P*) of each protein was calculated from the relative abundance (fmol/μg total protein) measured by LFQ multiplied by the molecular weight (MW; kDa) of the protein (referenced from the UniProt Knowledgebase). Absolute synthesis rates (ASR) were derived (Equation 4) by multiplying peptide *K*_*deg*_ by the absolute abundance of the protein at the end of the labelling period *P*(*t*).

(4) $$\mathrm{ASR}=P\left(t\right)⦁k_{\deg}$$

### 2.10 Statistical Analyses

With the exception of skeletal muscle proteome analysis, data were collated using Microsoft Excel. Statistical analyses were conducted using IBM SPSS Statistics (v. 29.0.0.0, IBM, Armonk, NY, USA) and figures were created using Prism (v. 10.4.1, Graphpad Software Inc., San Diego, California, USA). Anthropometric, resting metabolic rate, exercise indirect calorimetry data and changes in all blood‐borne markers were explored via linear mixed models. Significance was set at *p* < 0.05 for all statistical tests and data are reported as means ± SDs.

All statistical analysis of proteomic data was performed in R version 4.3.2 (2023‐10‐31). Within‐subject one‐way ANOVA was used to assess protein abundance and ASR. Differences in the abundance or ASR of proteins between EB and ED were reported as log_2_ transformed data and statistical significance was set at *p* < 0.05. A false discovery rate was not set, instead, *q* values [48] at the *p* = 0.05 were reported. Pairwise comparisons of the estimated marginal means for the experimental period were conducted using the Bonferroni adjustment for multiple comparisons in the linear mixed‐effects model with the emmeans R package [49].

### 2.11 Bioinformatic Analysis

Skeletal muscle proteomic data was processed and analyzed using R (Version 4.2.2) and figures were generated using the R package, ggplot2 [50]. The Search Tool for the Retrieval of INteracting Genes/proteins (STRING, Version 11.5) [51] was used to investigate protein interaction networks and calculate the enrichment of gene ontologies or KEGG pathways. Gene ontology analysis (GO) was performed via Overrepresentation Enrichment Analysis [52] using Gene Ontology enRIchment anaLysis and visuaLizAtion tool (Gorilla) [53] corrected against the experiment‐specific background consisting of all proteins that were included in statistical analysis. Enrichment of GO terms was considered significant if the Benjamin Hochberg adjusted *p* value was 0.01. Protein interaction networks were visualized by the Cytoscape string app [54] using the Omics Visualizer app [55]. The Mfuzz R package [56] was used to perform soft clustering analysis using the fuzzy c‐means clustering algorithm. The minimum membership value for inclusion into a cluster was set at 0.25. The coverage of mitochondrial Complex subunit proteins, fatty acid beta oxidation, and TCA cycle was survey as identified in Human MitoCarta 3.0 Rath et al. [57].

## 3 Results

Ten physically active, healthy men (participant characteristics are presented in Table 1) were studied during three consecutive 5‐day periods that were designed to manipulate energy availability and exercise (Figure 1A, further detailed in Appendix S1 (Protocol)). Participants were initially monitored during a lead‐in period of free‐living (FL; Days −5 to 0) when their diet and exercise were not controlled. The subsequent experimental periods included controlled exercise under either energy balance (EB; Day 0–5) or energy deficit (ED; Days 5–10) conditions. During EB, energy intake matched the requirements for the maintenance of body‐mass, whereas during ED each participant's energy intake and energy availability were controlled at 35% and 22% of the EB period, respectively (Table S1).

The participants' body mass (average 77.3 kg, standard deviation (SD) 8.1 kg) did not change during the FL and EB periods, whereas participants lost an average 2.98 kg (SD 0.7 kg; *p* < 0.001) after ED (Figure 1B). Body mass loss after ED included reductions in fat free mass (2.1 ± 0.8 kg; *p* < 0.001), fat mass (0.8 ± 0.6 kg; *p* < 0.001), and total body water (1.2 ± 1 kg; *p* < 0.001; Table 2).

**TABLE 2:** Body composition, blood parameters, skeletal muscle glycogen, and resting metabolic rate assessment.

	FL Day −5	FL | EB Day 0	EB Day 2	EB Day 4	EB | ED Day 5	ED Day 7	ED Day 9	ED Day 10	*p*	Days different from day 0 (*p* < 0.05)	Days different from day 5 (*p* < 0.05)
Body mass [kg]	77.3 (8.1)	77.5 (8.4)	79.7 (7.0)	78.1 (8.4)	77.6 (8.3)	76.1 (8.3)	75.2 (8.1)	74.6 (8.3)	< 0.001	2, 4, 7, 9, 10	2, 4, 7, 9, 10
Fat‐free mass—DXA—[kg]	64.8 (7.1)	65.4 (7.6)	—	—	65.1 (7.2)	—	—	62.9 (7.2)	< 0.001	−5, 10	10
Leg fat‐free mass—DXA—[kg]	10.6 (1.0)	10.8 (1.2)	—	—	10.7 (1.0)	—	—	10.4 (1.1)	< 0.001	10	10
Arms fat‐free mass—DXA [kg]	3.86 (0.45)	3.87 (0.47)	—	—	3.84 (0.49)	—	—	3.76 (0.49)	< 0.001	10	10
Fat mass—DXA—[kg]	13.8 (3.1)	13.8 (3.2)	—	—	14.0 (3.4)	—	—	13.2 (3.1)	< 0.001	10	10
Total bone mass—DXA‐[g]	2918 (322)	2919 (322)	—	—	2912 (320)	—	—	2935 (324)	0.47		
Total body water—BIA—[L]	47.8 (4.2)	47.8 (4.0)	49.4 (3.8)	48.6 (4.1)	48.0 (3.9)	47.1 (4.0)	47.0 (4.2)	46.8 (4.3)	< 0.001	2, 4, 7, 9, 10	2, 4, 7, 9, 10
Leptin [ng/mL]	1.75 (1.59)	1.65 (1.73)	1.97 (1.58)	1.92 (1.61)	1.44 (1.58)	1.07 (1.60)	1.02 (1.62)	0.76 (1.66)	< 0.001	2, 4, 7, 9, 10	2, 4, 7, 9, 10
T3 [ng/dL]	127 (29)	133 (33)	124 (26)	122 (31)	124 (27)	108 (27)	105 (27)	105 (26)	< 0.001	4, 7, 9, 10	7, 9, 10
IGF‐1 [ng/mL]	131 (54)	129 (53)	143 (65)	138 (56)	138 (54)	131 (51)	124 (61)	116 (49)	< 0.001	2, 10	9, 10
Testosterone [nmol/L]	20.2 (5.2)	21.6 (3.1)	21.8 (3.7)	22.9 (3.8)	21.4 (4.2)	22.3 (3.7)	21.8 (6.1)	20.4 (5.2)	0.39		
Insulin [ng/mL]	0.76 (0.33)	0.83 (0.52)	0.88 (0.50)	1.00 (0.83)	0.80 (0.54)	0.76 (0.49)	0.78 (0.52)	0.63 (0.50)	0.14		
EPO [mIU/mL]	8.8 (2.7)	8.7 (2.0)	—	—	9.8 (2.1)	—	—	9.3 (2.7)	0.54		
GDF‐15 [pg/mL]	387 (70)	379 (88)	350 (59)	363 (62)	342 (56)	374 (75)	388 (78)	363 (65)	< 0.001	2, 5	−5, 0, 7, 9
P1NP [μg/L]	82 (16)	80 (20)	81 (17)	79 (16)	79 (23)	72 (16)	69 (15)	67 (18)	< 0.001	7, 9, 10	7, 9, 10
β‐CTX [μg/L]	0.64 (0.23)	0.64 (0.24)	0.82 (0.25)	0.80 (0.29)	0.69 (0.21)	0.88 (0.19)	0.90 (0.23)	0.75 (0.21)	< 0.001	2, 4, 7, 9	2, 4, 7, 9
Glucose [mmol/L]	5.09 (0.59)	4.98 (0.58)	5.09 (0.45)	4.87 (0.47)	4.83 (0.30)	4.93 (0.42)	4.95 (0.40)	4.77 (0.44)	0.28		
β‐Hydroxybutyrate [mmol/L]	0.08 (0.02)	0.11 (0.08)	0.12 (0.09)	0.09 (0.05)	0.10 (0.08)	0.40 (0.31)	0.50 (0.31)	0.58 (0.36)	< 0.001	7, 9, 10	7, 9, 10
NEFA [mmol/L]	0.49 (0.34)	0.51 (0.20)	0.62 (0.27)	0.50 (0.44)	0.47 (0.36)	1.06 (0.39)	1.12 (0.49)	0.88 (0.39)	< 0.001	7, 9, 10	7, 9, 10
Glycerol [μmol/L]	30 (27)	20 (15)	30 (16)	29 (41)	25 (28)	62 (24)	78 (51)	51 (31)	< 0.001	7, 9, 10	7, 9, 10
Muscle glycogen [mmol/kg DM]	346 (139)	341 (74)	—	—	325 (83)	—	—	221 (70)	0.01	10	10
RMR [kcal/day]	1873 (246)	1856 (210)	—	—	1845 (247)	—	—	1755 (221)	0.15		
RMR [kcal/kg FFM/day]	29.0 (3.4)	28.5 (2.7)	—	—	28.3 (1.8)	—	—	28.0 (2.8)	0.69		
RMR fat oxidation [g/day]	108 (50)	116 (45)	—	—	81 (54)	—	—	138 (42)	0.006	5	10
RMR CHO oxidation [g/day]	219 (99)	195 (97)	—	—	282 (135)	—	—	109 (80)	< 0.001	5, 10	−5, 0, 10

*Note:* Values are means (standard deviation). N.B. The meals of each intervention phase started after the morning (fasted) measurements and exercise session of Days 0 and 5, therefore measurements of Days 0, 5, and 10 correspond to FL, EB, and ED diets, respectively.

Abbreviations: BIA, bioelectrical impedance analysis; CHO, carbohydrate; DM, dry mass; DXA, dual X‐ray absorptiometry; EB, energy balance period; ED, energy deficit period; EPO, erythopoietin; FFM, fat‐free mass; FL, free living period; GDF‐1, growth differentiation factor 1; IGF‐1, insulin‐like growth factor‐1; NEFA, non‐esterified fatty acids; P1NP, procollagen 1 N‐terminal propeptide; RMR, resting metabolic rate; T3, Triiodothyronine.

As per experimental design, during ED we observed the expected changes in endocrine and physiological blood markers associated with energy preservation in response to energy deficit, with metabolism switching from glucose to fat utilization at rest and during exercise, resembling previous reports [10, 18, 58] (Figure 1, and Figures S1 and S2; Tables 2 and 3). Specifically, during energy deficit there was a decrease in leptin, triiodothyronine, insulin‐like growth factor 1, and serum procollagen type I N‐propeptide (Figure 1C–F; Table 2). The decrease in energy and macronutrient availability produced a metabolic shift in circulating substrate availability evidenced by an increase in circulating β‐hydroxybutyrate concentrations and non‐esterified fatty acids (Figure 1G,H), and glycerol (Figure S1A) and a decrease in skeletal muscle glycogen content (Figure S1B), but no changes in glucose (Figure S1C) or insulin concentration (Figure S1D). Accordingly, there was an increase in fat oxidation after ED at rest (Figure 1I), and also during exercise (Figure 1J), mirrored by a decrease in carbohydrate oxidation at rest and during exercise (Figure S2A,B, respectively). We observed no changes in circulating testosterone, or EPO, a decrease in GDF‐15 during EB and similar pattern of β‐CTX increase during EB and ED (Figure S1E–H, respectively), and no changes in resting metabolic rate (Figure S2C,D).

**TABLE 3:** Exercise parameters.

Parameter	FL | EB Day 0	EB Day 2	EB Day 4	EB | ED Day 5	ED Day 7	ED Day 9	*p*	Days different from day 0 (*p* < 0.05)	Days different from day 5 (*p* < 0.05)
Total exercise time—net—[min]	92 (9.5)	92 (8.5)	93 (9.5)	92 (8.5)	92 (8.4)	93 (9.8)	0.91		
Exercise intensity [% V̇O_2_max]	63 (2)	63 (3)	62 (3)	63 (4)	63 (4)	63 (4)	0.90		
Exercise energy expenditure [kcal]	976 (104)	978 (109)	976 (107)	976 (105)	976 (106)	976 (106)	0.92		
Exercise energy expenditure [kcal/kg FFM]	15.1 (0.1)	15.1 (0.1)	15.0 (0.1)	15.1 (0.1)	15.1 (0.0)	15.1 (0.0)	0.53		
Exercise heart rate [BPM]	142 (10)	138 (11)	141 (5)	141 (7)	145 (8)	145 (9)	0.18		
Exercise RPE [AU 15‐point Borg scale]	12 (1)	12 (1)	12 (1)	12 (1)	12 (1)	12 (2)	0.55		
Exercise fat oxidation [g/min]	0.40 (0.13)	0.36 (0.09)	0.29 (0.08)	0.32 (0.08)	0.47 (0.08)	0.56 (0.10)	< 0.001	4, 5, 7, 9	0, 7, 9
Exercise CHO oxidation [g/min]	2.1 (0.4)	2.2 (0.6)	2.4 (0.6)	2.3 (0.5)	2.0 (0.5)	1.7 (0.4)	< 0.001	4, 7, 9	7, 9
Exercise gross efficiency at 50 W [%]	12.5 (1.9)	12.1 (1.6)	12.3 (1.7)	11.7 (1.2)	12.5 (1.5)	12.5 (1.5)	0.26		7, 9
Exercise gross efficiency at 75 W [%]	15.4 (1.8)	15.1 (1.5)	15.2 (1.3)	14.7 (1.2)	15.3 (1.2)	15.0 (1.3)	0.55		
Exercise gross efficiency at 100 W [%]	16.6 (1.4)	16.6 (0.7)	16.5 (1.1)	16.3 (1.0)	16.8 (1.1)	16.7 (1.0)	0.83		
Exercise gross efficiency at 125 W [%]	18.0 (1.0)	17.8 (0.7)	18.0 (1.0)	17.4 (0.9)	17.5 (0.9)	17.7 (0.9)	< 0.02	5	0, 4
Exercise gross efficiency during steady state [%]	17.1 (1.2)	17.0 (1.1)	17.1 (1.0)	17.0 (1.1)	16.9 (1.1)	17.0 (1.0)	0.99		

*Note:* Values are means (standard deviation). N.B. The meals of each intervention phase started after the morning (fasted) measurements and exercise session of Days 0 and 5, therefore measurements of Days 0, 5, and 9 correspond to FL, EB, and ED diets, respectively.

Abbreviations: AU, arbitrary units; BPM, beats per minute; EB, energy balance period; ED, energy deficit period; FFM, fat‐free Mass; FL, free living period; RPE, rate of perceived exertion.

### 3.1 Skeletal Muscle Mitochondrial Absolute Protein Synthesis Rates Are Up‐Regulated After the Energy Deficit Period

Across, FL, EB, and ED we measured absolute rates of protein synthesis of 736 proteins which were annotated in silico, and 388, 147, and 203 proteins were encompassed in soluble, myofibrillar, and mitochondrial annotation, respectively. There was no significant difference of average ASR in soluble (*p* = 0.17, Figure 1K) and myofibrillar (*p* = 0.13, Figure 1L) pools, but there was significant difference in the mitochondrial pool (*p* = 0.036, Figure 1M) represented by significantly faster ASR in ED compared to EB (*p* = 0.037). Detailed analysis of pathways regulated in protein ASR and abundance is shown in the following sections.

### 3.2 Proteomic Abundance and Absolute Synthesis Rates Show Distinct Responses After the Energy Deficit Period

**FIGURE 2:**
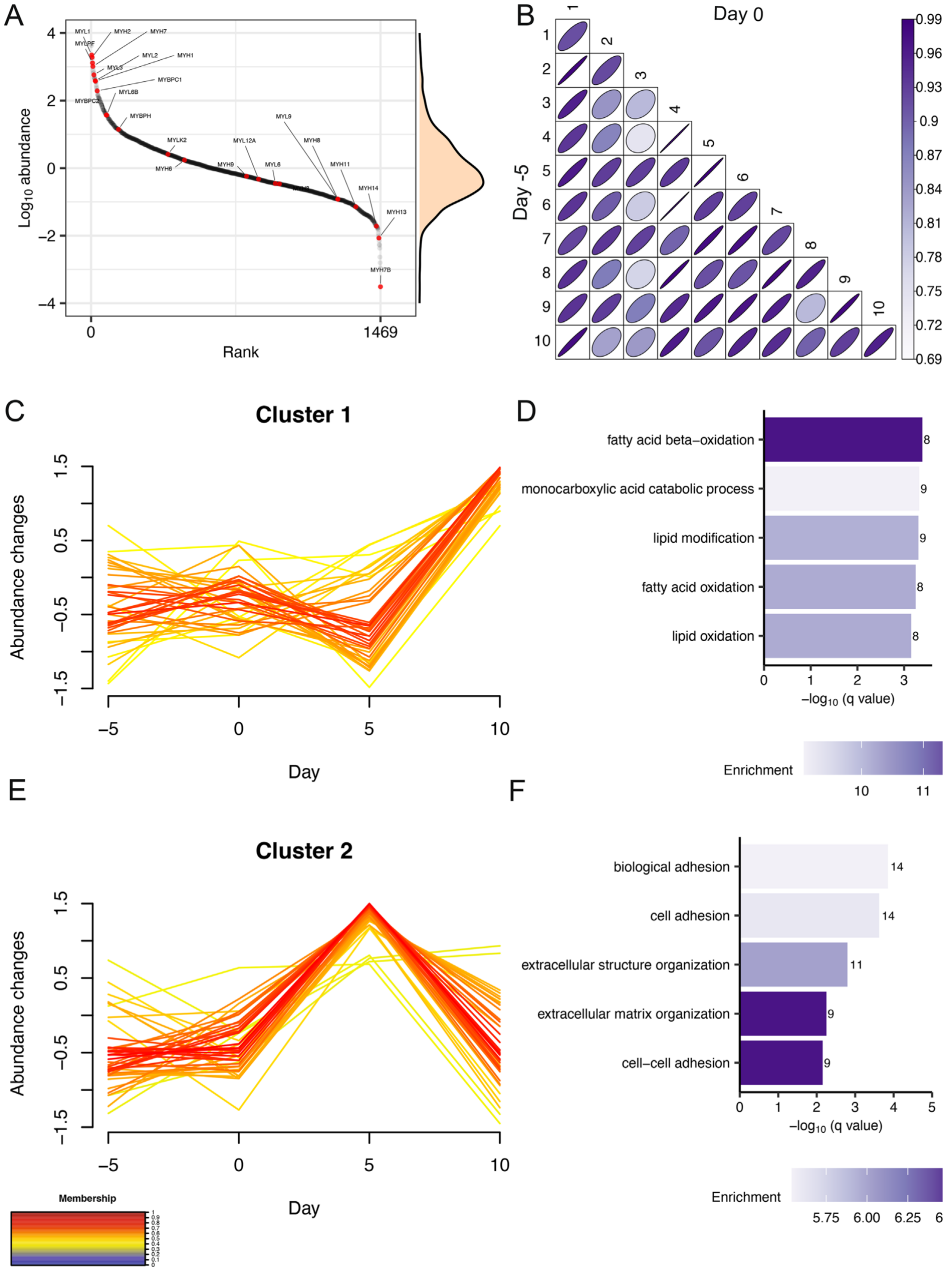
Unsupervised clustering analysis revealed two distinct time‐dependent changes in skeletal muscle proteome abundance across a 5‐day experimental period. (A) rank distribution plot of mean protein abundance between subjects at Day −5 (*n* = 1469 proteins). Red data points highlight high abundance of myosin‐related (muscle‐specific) proteins. (B) matrix correlation of abundance data between Day −5 and Day 0 within‐ and between‐subjects, *n* = 1469 proteins, using Pearson's correlation coefficient. C‐means fuzzy clustering separated 108 statistically significant proteins (within subject one‐way ANOVA *p* < 0.05) into 3 clusters with two major clusters; (C) Cluster 1 (*n* = 38) and (D) Cluster 2 (*n* = 44). Gene ontology (GO) analysis of Biological Process in proteins included in (E) Cluster 1 and (F) Cluster 2. GO terms were ranked by −log_10_ (q value) and the number of proteins included in each GO term reported alongside each entry. Each bar chart color scale represents the level of GO enrichment.

Proteomic analysis was conducted on 40 muscle samples taken at 4 time points (*n* = 10 participants) prior to, between and after the three experimental periods (Figure 1A, and Appendix S1 (Protocol)). Stringent filtering was used to ensure robust statistical analysis of the within‐subject repeated measures experimental design. Overall, 1671 proteins were confidently (FDR < 1%) identified, and after excluding proteins that were not detected in each of the four serial samples 1469 proteins had complete within‐subject data and were used for statistical analysis (Figure 2A). Protein abundance measurements were highly reproducible and the within‐subject coefficient of variation (CV) across the FL condition (Day −5 and Day 0) was M = 15.3% (interquartile range: 7%–30.8%, Figure 2B, Figure S4).

Time‐series analysis was conducted on protein abundance data and C‐means fuzzy clustering was used to discover shared temporal patterns amongst 108 proteins that exhibited significant (within‐subject ANOVA; *p* < 0.05, *q* < 0.41) differences across FL, EB, and ED conditions (Table S2). Unsupervised statistical methods, such as C‐means fuzzy clustering, are blind to the biological role of proteins, yet functional annotation of the resulting clusters found strong biological networks with supporting muscle‐specific literature on co‐function amongst proteins that shared temporal relationships (Figure 2D,F). Cluster 1 included proteins (*n* = 38) associated with mitochondrial metabolism that were unchanged during the FL and EB periods but significantly increased in abundance after 5 days ED (Figure 2C,D; Figure S6). Cluster 2 included proteins (*n* = 44) associated with the extracellular matrix, vesicles and exosomes that did not change during the FL period, increased during EB and decreased during ED (Figure 2E,F; Figure S7). Proteins (*n* = 26) included in Cluster 3 exhibited heterogenous patterns of change, were less strongly clustered (had lower membership values) and did not include well‐defined biological relationships (Figure S8).

**FIGURE 3:**
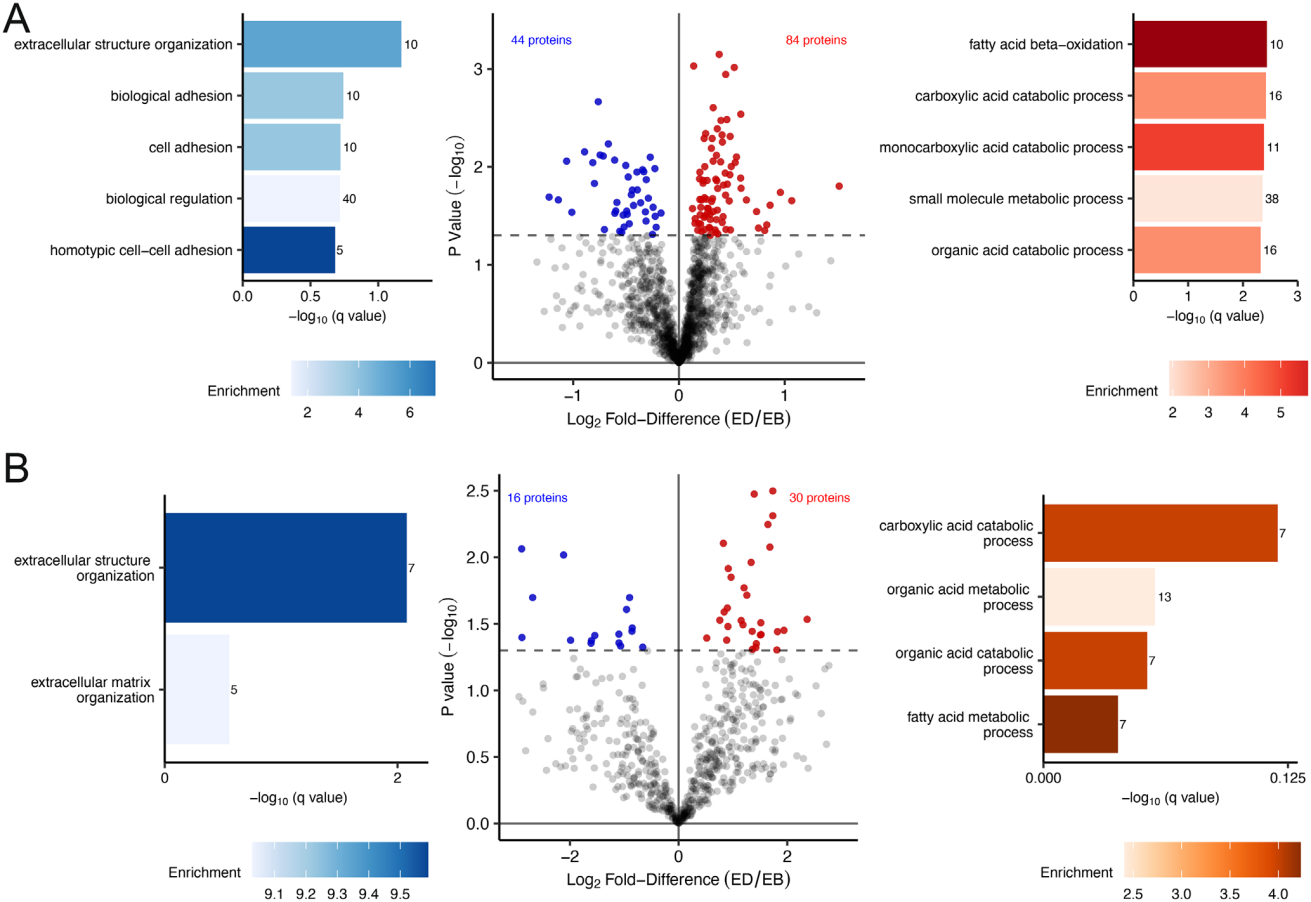
Energy deficit increased the abundance and absolute synthesis rates of proteins of mitochondrial metabolic processes and decreased the abundance and absolute synthesis rates of connective tissue. (A) volcano plot comparing the Log_2_ Fold‐Difference (ED/EB) protein abundance plotted against the −Log_10_ *p* value (*n* = 1469). Colored data points represent proteins significantly more abundant (red, log_2_ Diff. > 0 and *p* < 0.05), less abundant (blue, log_2_ Diff. < 0 and *p* < 0.05), or stable (gray, *p* > 0.05) in ED compared to EB. (B) volcano plot comparing the Log_2_ Fold‐Difference (ED/EB) ASR plotted against the −Log_10_ *p* value (*n* = 625). Colored data points represent proteins significantly faster ASR (red, log_2_ Diff. > 0 and *p* < 0.05), slower ASR (blue, log_2_ Diff. < 0 and *p* < 0.05), or stable (gray, *p* > 0.05) in ED compared to EB. The dashed horizontal line shows a threshold of statistical significance (*p* < 0.05) without corrections for multiple comparison. Gene ontology (GO) analysis of Biological Process in proteins significantly different between ween ED and EB. GO terms were ranked by −log_10_ (*q* value) and the number of proteins included in each GO term reported alongside each entry. Each bar chart color scale represents the level of GO enrichment.

Overall, the majority (93%) of proteins were unaltered in abundance across FL, EB, and ED periods with highly reproducible proteome across the FL condition (Day −5 and Day 0). Coordinated changes to the protein abundance profile of human muscle during the ED period were compared against EB period. There were 128 differences (*p* < 0.05, *q* < 0.387) between EB and ED, including 84 proteins that were more abundant and 44 proteins that were less abundant in ED compared to EB (Figure 3A, Table S3). GO terms that were significantly enriched amongst proteins that were more abundant in ED, included mitochondrion (GO:0005739, q = 1.27E‐08), fatty‐acid beta‐oxidation (GO:0006635, *q* = 0.0037), carboxylic acid catabolic processes (GO:0046395, *q* = 0.0038), monocarboxylic acid catabolic processes (GO:0072329, *q* = 0.0041), small molecule metabolic processes (GO:0044281, *q* = 0.0044), and organic acid catabolic process (GO:0016054, *q* = 0.0048) (Figure 3A). Whereas, the GO term, extracellular structure organization (GO:0043062, *q* = 0.068), was enriched amongst proteins that were significantly less abundant in ED included (Figure 3A).

Protein‐specific synthesis rates were investigated by biosynthetically labelling newly synthesized proteins using the stable isotope, deuterium oxide (D_2_O). Participants consumed D_2_O throughout the study period and a steady state enrichment of 0.84% ± 0.11% of the body water compartment was achieved. Body water enrichment remained stable within each experimental period, and body water enrichment was not different between FL, EB, and ED (*p* = 0.934). Body water enrichment during EB and ED periods were 0.836% ± 0.106% and 0.840% ± 0.126%, respectively. In the calculation of protein turnover, the precursor enrichment of each participant during each experimental period was accounted in the Equation (2). The absolute synthesis rate (ASR, ng/μg total protein) of individual muscle proteins was calculated from high‐quality peptide mass isotopomer data collected from muscle samples that spanned each experimental condition. ASR data portrays both the rate of synthesis and abundance of each protein, which is essential to interpret differences in protein synthesis between muscles with different protein abundance profiles. Stringent filtering was used to ensure robust statistical analysis of 736 proteins that had complete protein synthesis data across FL, EB, and ED conditions.

To provide an overview, protein‐specific ASR data were summed to reflect the bulk (mixed‐protein) synthesis rate among myofibrillar, mitochondrial, and sarcoplasmic muscle components. The human MitoCarta database was used to identify (*n* = 203) mitochondrial proteins, the selection of myofibrillar (*n* = 147) proteins was based on a manually curated subset of GO:0030016 and the remaining (*n* = 388) proteins were regarded as being sarcoplasmic. The sum ASR of mitochondrially annotated proteins was 47% greater (*p* = 0.037) during ED (25.95 ± 7.42 ng/μg total protein/day) than EB (17.61 ± 3.51 ng/μg total protein/day), whereas there were no significant differences in the synthesis rate of mixed myofibrillar (515 ± 169 ng/μg total protein/day) or sarcoplasmic (71 ± 49 ng/μg total protein/day) proteins throughout the FL, EB, and ED conditions (Figure 1K–M). Increased ASR after ED in mitochondrial annotation is in line with increased FSR (*p* = 0.074, Figure S3) and abundance (Figures 2A and 3A) specifically in mitochondrial annotation.

The more detailed effects of ED on skeletal muscle protein turnover were investigated by comparing protein‐specific ASR data between EB and ED conditions. There were 46 differences (*p* < 0.05, *q* < 0.30) between EB and ED, including 30 proteins with higher ASR and 16 proteins that with lower ASR in ED as compared to EB (Figure 3B, Table S4). GO terms, including extracellular structure organization (GO:0043062, *q* = 0.0083) and extracellular matrix organization (GO:0030198, *q* = 0.28) were enriched among proteins with lower ASR during ED. Proteins that increased in ASR, included MYH7 and enzymes of fatty‐acid beta‐oxidation but GO terms were not statistically enriched amongst these proteins. MYH7 is a slow isoform of myosin heavy chain, expressed in slow‐twitch oxidative muscle fibers, and the gain in both the abundance and synthesis rate of MYH7 during ED is consistent with studies on weight loss without exercise, reporting increases in muscle MYH7 mRNA expression alongside lower circulating levels of leptin and T3 [24, 59].

### 3.3 Enhanced Muscle Mitochondrial Adaptations After the Energy Deficit Period

**FIGURE 4:**
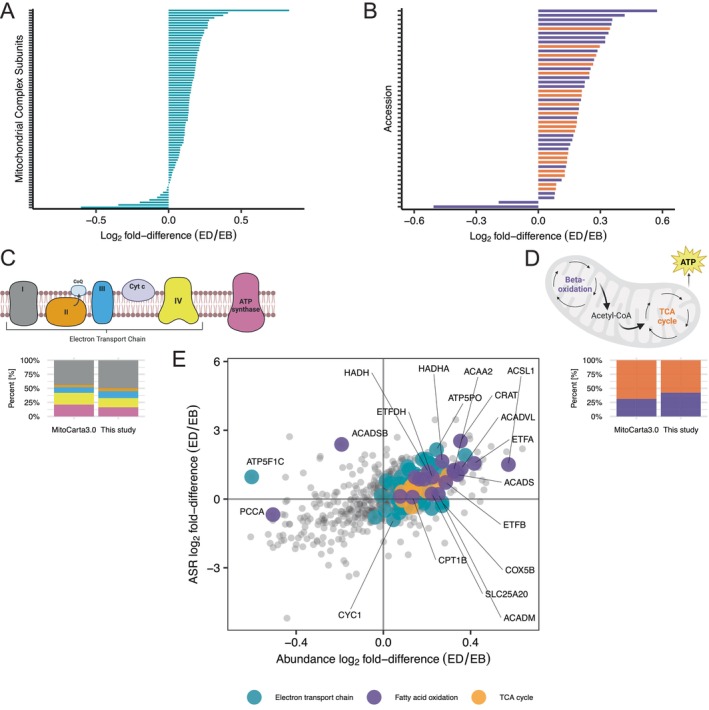
Energy deficit induces increase in abundance and ASR of proteins related to electron transport chain, fatty acid beta oxidation, and TCA cycle. Bar chart comparing the Log_2_ Fold‐Difference (ED/EB) protein abundance of (A) electron transport chains and (B) fatty acid beta oxidation and TCA cycle. Stacked bar chart shows the relative distribution of mitochondrial proteins contained in and classified by the Human MitoCarta3.0 database for electron transport chain (C) and Beta‐oxidation and TCA cycle (D). (E) Scatter plot comparing the differences in the Log_2_ Fold‐Difference (ED/EB) between protein abundance (*x*‐axis) and protein ASR (*y*‐axis). Proteins related to electron transport chain, fatty acid beta oxidation, and TCA cycle are colored by blue, purple, and orange, respectively. Shiny app can be accessed via 10.6084/m9.figshare.27169632.

Proteome profiling encompassed the majority of respiratory chain subunits and enzymes of the TCA cycle and fatty acid beta‐oxidation which showed predominant increase in the abundance of proteins assessed (Figure 4A–D). A common pattern of increases in the abundance and synthesis was evident across mitochondrial proteins specifically during ED (Figure 4E). The TCA cycle enzyme, citrate synthase (CS), is a commonly used biomarker of mitochondrial metabolism [23, 60] and was amongst the array of mitochondrial proteins that increased during ED (Figure 3 and Figure 4). Our findings agree with the effects of large‐magnitude weight loss (7%–21% body mass) achieved through energy deficit and exercise in people with obesity, which reported increases in mitochondrial respiratory capacity [23] and mitochondrial enzyme activity, including CS [23, 60], though are at odds with the finding of Caldwell et al. [18] showing no changes in CS activity in trained females following 14 days of low energy availability. The mitochondrial adaptations to ED cooccurred with multilevel evidence of changes in lipid metabolism, including shifts increases in fat oxidation at rest and during exercise (Figure 1I,J), circulating levels of non‐esterified fatty acids (Figure 1H), and ketones (Figure 1G) and muscle perilipin (PLIN) proteins (Figure S5), which are integral to the storage and breakdown of intramuscular triglyceride‐containing lipid droplets [61].

ED was associated with increases in the abundance of PLIN2 and PLIN5 and an increase in the ASR PLIN4. The discovery proteome abundance data were verified by orthogonal analysis using targeted mass spectrometry, which quantified PLIN2 and PLIN5 against stable isotope‐labeled internal standards and verified both PLIN2 and PLIN5 significantly increased after ED (Figure S5). Exercise training in humans increases the content of both PLIN2 and 5 proteins [62, 63], which is likely to be an important adaptation to support greater intramuscular triglyceride storage, especially in the face of elevated free fatty acid availability produced by ED (Figure 1H). Furthermore, PLIN5 is proposed to create a physical and metabolic link between lipid droplets and mitochondria, facilitating triglyceride hydrolysis and fatty acid oxidation [64].

PLIN4 is the most abundant perilipin in muscle but the role of PLIN4 in muscle metabolic responses to exercise is not yet clear. PLIN4 is primarily located to sarcolemma regions of slow twitch fibers [65] and in adipocytes, PLIN4 coats nascent lipid droplets earlier than other PLIN proteins [66]. The muscle mRNA expression of PLIN4 decreases after 12 weeks of training [65], whereas we [67] have reported a trend for greater PLIN4 protein abundance in the muscle of endurance‐trained athletes. Our finding that PLIN4 synthesis increases without any change in PLIN4 abundance, together with the earlier observations, suggests the response of muscle PLIN4 to exercise involves a change in turnover rate rather than change in protein abundance.

Key proteins associated with mitochondrial quality, including LONP1, PITRM1, SSBP1, TIMM13, TIMM9, BDH1, and ALDH2 were enriched and had higher rates of synthesis during the ED compared to EB period. Elevations in BDH1, which is a component of muscle ketone body metabolism, link with the increased levels of circulating ketones (Figure 1G) and heightened fat utilization at rest (Figure 1I) during exercise (Figure 1J) under the ED condition. In mice, ketone flux through BDH1 is essential to the adaptive response to intermittent time‐restricted feeding (iTRF), and similar to our ED condition in humans, iTRF in mice increases muscle TCA cycle, OXPHOS and ETC protein abundance [68].

Muscle‐specific knockout of BDH1 disrupts mitochondrial remodeling and is associated with lesser abundance of LONP1 [68], which agrees with our observations of parallel increases in BDH1 and LONP1 in human muscle during ED conditions. LONP1 is a mitochondrial protease involved in the activation of PINK1/Parkin pathway [69] and, in mice, treadmill exercise increases LONP1 abundance alongside other markers of mitochondrial unfolded protein response [70]. High‐intensity aerobic training in humans [71] did not change mitochondrial LONP1 abundance, which may point to a specific benefit of the ED intervention in humans.

The abundance of the mitochondrial matrix peptidase, PITRM1, also increased during ED and is positively associated with mitochondrial function. Humans with loss‐of‐function mutations of PITRM1 have markedly less active muscle respiratory chain complexes and, in mice, lower PITRM1 content is associated with lower levels of O_2_ utilization [72]. The benefits of exercise during ED extended to other mitochondrial protective mechanisms, including aldehyde dehydrogenase (ALDH2), which is an antioxidant enzyme that protects against lipid‐peroxidation modifications to proteins. ALDH2 is more abundant in the muscle of high‐capacity runner rats [73], which are protected from cardiometabolic disease and muscle dysfunction. Moreover, overexpression of ALDH2 restores mitochondrial dysfunction, attenuates levels of muscle atrophy markers, and enhances physical capacity in mice ([74]). Proteins associated with mitochondrial DNA stability, including SSBP1 [75] and MSH5 [76] also had higher levels of synthesis and abundance during ED and add evidence on the positive effects on mitochondria.

### 3.4 The Skeletal Muscle Extracellular Matrix and Cytoskeleton Proteins Evidence Complex Modulation During the Energy Deficit Period

**FIGURE 5:**
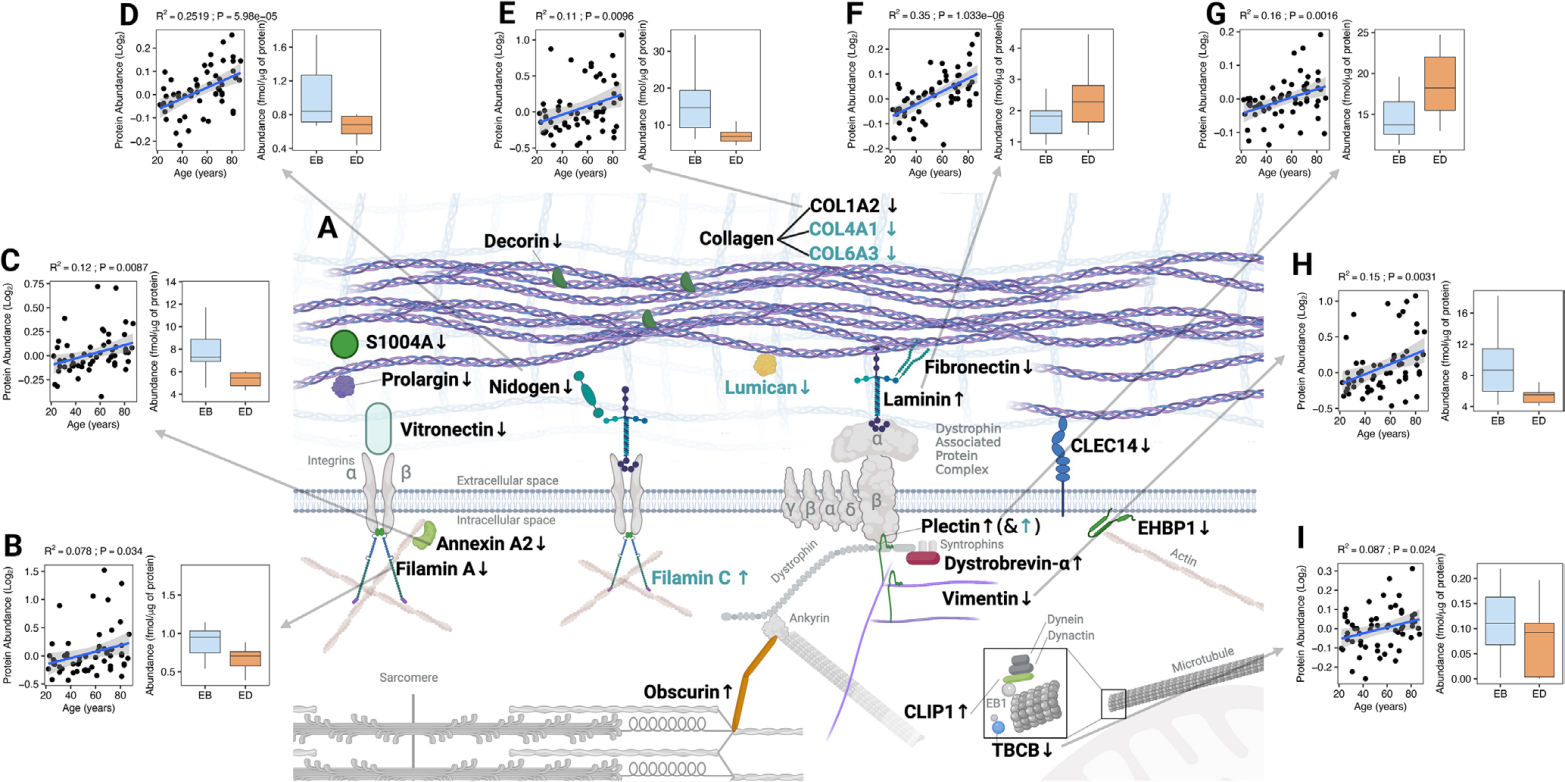
Schematic representation of extracellular matrix and cytoskeletal protein abundance (black text) and absolute synthesis rate (ASR; light emerald green text) changes in response to energy deficit (A), and relationship between abundance of proteins and aging data generated by Ubaida‐Mohien et al. [77], compared to changes in abundance in our study (panels B–I). The majority of the proteins associated to the extracellular matrix (collagen—COL1A2, COL4A1, and COL6A3—, decorin, fibronectin, nidogen, vitronectin, prolargin, S100A4, and CLEC14A), as well as those of the intracellular space associated to the cytoskeleton (annexin, filamin A, EHBP1L1 and vimentin) were reduced in abundance or ASR during the energy deficit period. In contrast, proteins associated to the dystrophin associated protein complex (Laminin, dystrobrevin‐α, plectin, and obscurin) were increased. An exception is CLIP1, which may be related to an increase in stability of the polymerization on the plus end of microtubules, and Filamin C. Proteins FLNA (B), ANXA2 (C), NID1 (D), COL1A2 (E), LAMA2 (F), PLEC (G), VIM (H), and TBCB (F) and showed an abundance positively correlated with aging [77], a trend that was opposed by changes in abundance in COL1A2, NID1, FLNA, TBCB, VIM, and ANXA2, but not LAMA2 or PLEC. Colored proteins are proteins for which we report significant changes, light‐gray/faded proteins are added for context. Figure created in Biorender (https://BioRender.com/bjrrb8m).

Proteins with roles in extracellular structure and organization exhibited complex patterns of change that suggest reorganization of the cytoskeleton and ECM during ED (Figures 3A and 5 and Figure S9). Decreases in the abundance and ASR of extracellular matrix‐linked proteins (COL1A2, DCN, FN1, PRELP, S100A4, VTN, AZGP1, NID1, CLU, CLEC14, APOA1 and AGT, COL4A1, COL6A3, and LUM), and cytoskeletal proteins (ANXA2, EHBP1L1, FLNA, TBCB, and VIM) cooccurred with increases in abundance and ASR of proteins associated with the dystrophin‐associated protein complex (DPC) (DTNA, PLEC, LAMA2, and OBSCN) and tubulin polymerization (CLIP1).

Collagen is a major component of the extracellular matrix [78] and was particularly responsive to ED. Fibrillar collagen (COL1A2), network collagen (COL4A1 and COL6A3) and proteins with roles in collagen binding and genesis (DCN, S100A4, PRLEP, NID1, FN1, VTN and CLEC14A, and LUM) were less abundant or had lower rates of synthesis after ED. The decreases in collagen abundance and synthesis are consistent with the changes in circulating markers of collagen formation (P1NP, Figure 1F) and collagen breakdown (β‐CTX, Figure S1H) in ED. Furthermore, lumican decreased in ASR during ED and is a key extracellular matrix proteoglycan [79] and a myokine with effects on bone formation [80]. The decrease in ASR of lumican cooccurred with decreases in abundance of ECM glycoproteins (decorin, vitronectin, and nidogen), and mixed responses amongst basement membrane and cytoskeletal proteins. Laminin (LAMA2) is an important component of the basement membrane and increased in abundance, whereas the abundance of the basement membrane protein, prolargin, decreased. LAMA2 interacts with integrins and alpha‐dystroglycan, and components of the DPC, including DTNA, OBSCN, and PLEC also increased in abundance during ED. Other components of the DPC (SNTA1, SNTB1, DAG1, and DMD) were included in the proteomic data but did not change in abundance, and proteins with roles in the actin cytoskeleton (EHBP1L1, FLNA, and ANXA2), intermediate filaments (VIM and FLNC), and microtubule network (CLIP1 and TBCB) were also responsive to ED.

Aging is associated with an accumulation of extracellular matrix proteins in muscle [33, 81] that, in rhesus monkeys, can be countered by life‐long caloric restriction [35, 81]. We investigated whether the muscle responses to ED reported here in young adults oppose the effects of aging on extracellular matrix proteins across human adult aging. To do this we conducted a secondary analysis of the data generated by Ubaida‐Mohien et al. [77], which showed a significant positive relationship between aging and the abundance of ECM proteins (FLNA, ANXA2, NID1, COL1A2, LAMA2, PLEC, VIM, and TBCB) in skeletal muscle. We found six (FLNA, ANXA2, NID1, COL1A2, VIM, and TBCB) out of eight of these proteins exhibited the opposite effect (decreased in abundance) after ED (Figure 5B–F). Care should be taken in the comparison of our data (Figure 5A) with those generated in the study by Ubaida‐Mohien et al. [77] (Figure 5B–I) given that it represents a comparison between a short‐term intervention and cross sectional differences across adult lifespan. Nonetheless, these contrasting effects of aging and energy deficit on the human muscle proteome add support to the experimental observations on life‐long energy deficit in rhesus monkeys [35, 81].

## 4 Discussion

Skeletal muscle is central to metabolic regulation and locomotion, yet the impact of acute energy deficit on muscle phenotype in exercising humans remains poorly defined. We show that after a 5‐day period of pronounced energy restriction (78% reduction in energy availability), combined with aerobic‐type exercise, skeletal muscle proteome in young healthy adults shows a phenotype change. This remodeling involves a shift toward a more oxidative phenotype, with increased synthesis of mitochondrial proteins and elevated abundance of enzymes involved in fatty acid β‐oxidation, the tricarboxylic acid cycle, electron transport, and mitochondrial quality control, alongside enhanced lipid storage. Notably, these adaptations occurred without reductions in bulk myofibrillar or sarcoplasmic absolute protein synthesis. To the best of our knowledge, we are also the first to report decreased abundance of extracellular matrix proteins and increased levels of components of the dystrophin‐associated protein complex in human skeletal muscle following energy deficit. These coordinated proteomic responses suggest an improvement in muscle quality and metabolic efficiency, with potential implications for interventions aimed at enhancing healthspan, lifespan, and physical capacity.

Our data offers new molecular insights into the mitochondrial adaptations following energy deficit overlayed on top of an exercise stimulus in humans. Consistent with studies involving substantial weight loss of overweight or obese individuals—who concomitantly undertook physical exercise—that show increases in the content and activity of some mitochondrial enzymes [23, 82], we observed a coordinated increase in mitochondrial protein synthesis resulting in upregulation of a broad array of mitochondrial proteins. Notably, 287 out of 343 quantified mitochondrial proteins were increased in abundance, reflecting widespread remodeling of the mitochondrial proteome. Prominent protein networks regulated after the energy deficit period were associated with an enhancement of mitochondrial proteostasis, mtDNA‐quality control, and protein import, which agree with transcriptomic responses reported in humans following 2 years of 12% caloric restriction with no exercise [8]. A possible key candidate we identify mediating this response is BDH1, which has been linked to increases in fatty acid oxidation efficiency and exercise capacity with intermittent‐time‐restricted feeding‐induced energy deficit in mice [68]. These findings support a synergistic role of energy restriction and exercise in promoting mitochondrial biogenesis and are aligned with murine data showing increased mitochondrial content and improved physical capacity [34]. Improvements in mitochondrial metabolism by energy deficit have also been linked to the maintenance of a youthful skeletal muscle phenotype in life‐long energy restricted macaque monkeys, reflected in a reduction in the age‐related decline of muscle contractile area resulting from fibrosis [35], which is aligned with our observations of the extracellular matrix and cytoskeletal protein response.

Our results also point to notable changes in ECM and cytoskeletal proteins, which may be relevant for muscle aging and fibrosis. Skeletal muscle fibrosis is a hallmark of aging [33, 83], and lifelong caloric restriction has been shown to prevent fibrotic accumulation in monkeys [35, 81]. Here, we present the first human data indicating that after the short‐term energy deficit period there is a reduction of ECM proteins associated with aging, including COL1A2 and nidogen, as well as others such as decorin, S100A4, prolargin, vitronectin, fibronectin, and CLEC14. In contrast, proteins associated with the dystrophin‐associated protein complex (e.g., laminin, plectin, dystrobrevin‐alpha, and obscurin) increased in abundance, suggesting enhanced membrane stability and cell adhesion in response to ECM remodeling. These findings raise the possibility that energy deficit could therapeutically reduce pathological ECM accumulation linked to impaired glucose metabolism and sarcopenia [84, 85].

The modulation of ECM proteins during ED aligns with recent plasma proteome data from individuals undergoing prolonged fasting, where extracellular proteins were enriched [86]. The downregulation of non‐essential structural proteins after the energy deficit period, combined with endocrine adaptations such as reduced T3, leptin, and IGF‐1, and enhanced fat oxidation and aerobic metabolic pathway activity, aligns with trade‐offs predicted by life‐history theory [3], whereby there may be a reallocation of energetic resources for processes that are immediately important for survival. In this context, our findings suggest that during energy‐limited states, skeletal muscle prioritizes the maintenance of contractile and metabolic capacity—particularly when stimulated by exercise. This interpretation of the findings provides a conceptual framework to further enquire about the potential functional and health effects of GLP‐1 agonist‐induced weight‐loss on skeletal muscle in the context of the current debate [15, 16]. Our findings highlight the importance of looking beyond reductions in bulk protein synthesis and muscle size with energy deficit and for future research to focus on metabolic, non‐metabolic, and functional changes of skeletal muscle to understand the potential positive and negative effects of energy deficit on muscle and overall health.

Our study has limitations. The quasi‐experimental design restricts causal inference. Although the energy balance (EB) period, used as a comparison, included a controlled diet and an exercise stimulus familiar to the participants, the fixed order of the periods (EB followed by ED) prevents us from fully ruling out a time‐effect influence in some of our observations. The small sample size (*n* = 10), consisting solely of healthy young males, limits generalizability. While participants maintained perceived effort during exercise, we did not formally assess physical performance. The intervention's short duration and severity provided a useful model to detect molecular changes, but future research should examine clinically relevant protocols. Although macronutrient intake was standardized as a percentage of total energy intake, varying protein levels could have affected protein synthesis [17, 87]. Our sample size is comparable to or greater than most previous studies [30, 88, 89, 90] employing stable isotope labeling (D_2_O) and peptide mass spectrometry to measure muscle protein synthesis at the individual protein level. Nevertheless, our proteomic data may still be underpowered for detecting effects of smaller magnitude. Future dynamic proteome profiling studies should therefore aim for longer intervention periods and larger participant numbers to increase the sensitivity for detecting biologically meaningful effects of energy deficit.

In conclusion, our findings reveal a dynamic skeletal muscle proteome response following short‐term energy deficit combined with exercise, characterized by enhanced mitochondrial remodeling and reductions in extracellular matrix proteins. These adaptations suggest a shift toward improved metabolic health and resistance—of the variables measured—to muscle aging, even in the face of hormonal and metabolic signals favoring energy conservation. Such responses likely reflect a conserved evolutionary strategy for maintaining physical capacity under energetic constraint. As energy‐restricted states become increasingly relevant due to lifestyle, medical, and pharmacological interventions, understanding their effects on muscle health is critical. Our work offers a molecular foundation for future studies aimed at leveraging energy deficit to optimize skeletal muscle function and overall health.

## Author Contributions

J.L.A. was involved in conceptualization. Y.N., C.L.‐E., H.L.T., J.G.B., and J.L.A. were involved in data curation/software. Y.N., C.L.‐E., H.L.T., J.G.B., and J.L.A. were involved in formal analysis. Y.N., H.L.T., W.L.F., J.A.S., S.S., J.G.B., and J.L.A. were involved in methodology. Y.N. and J.L.A. were involved in visualization. J.G.B. and J.L.A. were involved in funding acquisition. J.L.A. was involved in project administration. C.L.‐E., J.P.M., J.G.B., and J.L.A. were involved in supervision. Y.N., J.G.B., and J.L.A. were involved in writing original draft. Y.N., C.L.‐E, H.L.T.,W.L.F., J.P.M, J.A.S., S.S., J.G.B., and J.L.A. were involved in writing review and editing.

## Conflicts of Interest

The authors declare no conflicts of interest.

## Supporting information


**Figure S1:** Blood metabolites and hormones and skeletal muscle glycogen. Change in (A) plasma glycerol, (B) Skeletal muscle glycogen, (C) plasma glucose, (D) plasma insulin, (E) serum testosterone, (F) serum erythropoietin, (G) serum GDF‐15, and (H) plasma β‐CTX. Absolute values and statistical analyses are presented in Table 2. *n* = 10 (individuals) × 4 or 8 (samples).
**Figure S2:** Resting and exercise‐related respiratory parameters. Change in (A) Resting carbohydrate (CHO) oxidation at rest, (B) CHO oxidation during exercise, (C) Resting metabolic rate and (D) Resting metabolic rate (relative to fat free mass). Absolute values and statistical analyses are presented in Table 2. Details of exercise variables are presented in Table 3. *n* = 10 (individuals) × 4 or 6 (samples).
**Figure S3:** Fractional synthetic rate (%/day) of proteins annotated into soluble, myofibrillar, and mitochondrial assessed in silico. The box plot represents the interquartile range (IQR; 25th–75th percentile), with the horizontal line indicating the median. Whiskers extend to the minimum and maximum values within 1.5× IQR. FSR (%/day) values for individual subjects are shown as black data points. Gray lines connect values from the same subject across the three periods (FL, EB, and ED). The red diamond represents the mean. Pairwise comparisons of the estimated marginal means for the experimental period were conducted using the Bonferroni adjustment for multiple comparisons in the linear mixed‐effects model.
**Figure S4:** Skeletal muscle proteome abundance is stable during the FL condition. (A) a scatter plot of protein abundance data between Day −5 and Day 0 from all 10 participants. (B) violin plots of Coefficient of Variation between Day −5 and Day 0 on a protein‐by‐protein basis.
**Figure S5:** Targeted proteomic analysis via parallel reaction monitoring verifies PLIN2 and PLIN5 are more abundant in energy deficit. Box plot illustrating absolute protein abundance of (A) PLIN2 and (B) PLIN5. A scatter plot comparing absolute protein abundance between two independent injections into LC‐MS in (C) PLIN2 and (D) PLIN5. Coefficient of variation (CV) of absolute protein abundance between two independent injections into LC‐MS in E, PLIN2 and F, PLIN5.
**Figure S6:** Box plots of proteins in cluster 1 identified by c‐means fuzzy clustering. Box plots illustrating changes of protein abundance across the experimental period (38 proteins, all *p* < 0.05). The box plot represents the interquartile range (IQR; 25th–75th percentile), with the horizontal line indicating the median. Whiskers extend to the minimum and maximum values within 1.5× IQR.
**Figure S7:** Box plots of proteins in cluster 2 identified by c‐means fuzzy clustering. Box plots illustrating changes of protein abundance across the experimental period (44 proteins, all *p* < 0.05). The box plot represents the interquartile range (IQR; 25th–75th percentile), with the horizontal line indicating the median. Whiskers extend to the minimum and maximum values within 1.5× IQR.
**Figure S8:** Box plots of proteins in cluster 3 identified by c‐means fuzzy clustering. Box plots illustrating changes of protein abundance across the experimental period (26 proteins, all *p* < 0.05). The box plot represents the interquartile range (IQR; 25th–75th percentile), with the horizontal line indicating the median. Whiskers extend to the minimum and maximum values within 1.5× IQR.
**Figure S9:** STRING network of proteins belonging to the extracellular matrix and cytoskeletal network identified through GO network analyses and independent analysis of significantly different proteins between EB and ED (Table S3). The boarder color represents Log_2_ fold‐difference in protein abundance between ED and EB.

**Table S1:** Nutritional intake.

**Table S2:** Statistical output of within‐subject one‐way ANOVA in protein abundance and 108 statistically significant proteins with cluster and membership value—related to Figure 2.

**Table S3:** Statistical output of within‐subject one‐way ANOVA in protein abundance—related to Figure 3A.

**Table S4:** Statistical output of within‐subject one‐way ANOVA in ASR—related to Figure 3B.

**Appendix S1:** fsb271163‐sup‐0006‐AppendixS1.docx.

## Data Availability

The mass spectrometry proteomics data generated in this study have been deposited to the ProteomeXchange Consortium via the PRIDE [91] partner repository with the dataset identifier PXD061267 and 10.6019/PXD061267. Raw files of parallel reaction monitoring, Skyline document, processed results used as input for figure generation are available on PanoramaWeb [92] (https://panoramaweb.org/margulis2025.url) and ProteomeXchange with the identifier PXD061311. R script and raw data used to generate the Shiny app were deposited in figshare (DOI: 10.6084/m9.figshare.27169632).
