# Supplementary material for: Endocrine, Metabolic, and Skeletal Muscle Proteomic Responses During Energy Deficit With Concomitant Aerobic Exercise in Humans
Source: FASEB J. 2025 Nov 3;39(21):e71163. doi: 10.1096/fj.202502384RR (PMC12582342; doi:10.1096/fj.202502384RR)
Supplement: Supplementary file 1 — Figure S1: Blood metabolites and hormones and skeletal muscle glycogen. Change in (A) plasma glycerol, (B) Skeletal muscle glycogen, (C) plasma glucose, (D) plasma insulin, (E) serum testosterone, (F) serum erythropoietin, (G) serum GDF‐15, and (H) plasma β‐CTX. Absolute values and statistical analyses are presented in Table 2. n = 10 (individuals) × 4 or 8 (samples). Figure S2: Resting and exercise‐related respiratory parameters. Change in (A) Resting carbohydrate (CHO) oxidation at rest, (B) CHO oxidation during exercise, (C) Resting metabolic rate and (D) Resting metabolic rate (relative to fat free mass). Absolute values and statistical analyses are presented in Table 2. Details of exercise variables are presented in Table 3. n = 10 (individuals) × 4 or 6 (samples). Figure S3: Fractional synthetic rate (%/day) of proteins annotated into soluble, myofibrillar, and mitochondrial assessed in silico. The box plot represents the interquartile range (IQR; 25th–75th percentile), with the horizontal line indicating the median. Whiskers extend to the minimum and maximum values within 1.5× IQR. FSR (%/day) values for individual subjects are shown as black data points. Gray lines connect values from the same subject across the three periods (FL, EB, and ED). The red diamond represents the mean. Pairwise comparisons of the estimated marginal means for the experimental period were conducted using the Bonferroni adjustment for multiple comparisons in the linear mixed‐effects model. Figure S4: Skeletal muscle proteome abundance is stable during the FL condition. (A) a scatter plot of protein abundance data between Day −5 and Day 0 from all 10 participants. (B) violin plots of Coefficient of Variation between Day −5 and Day 0 on a protein‐by‐protein basis. Figure S5: Targeted proteomic analysis via parallel reaction monitoring verifies PLIN2 and PLIN5 are more abundant in energy deficit. Box plot illustrating absolute protein abundance of (A) PLIN2 and (B) PLIN5. A scatter plot [file FSB2-39-e71163-s004.docx]

**Figure S1.** Blood metabolites and hormones and skeletal muscle glycogen. Change in **A)** plasma glycerol, **B)** Skeletal muscle glycogen, **C)** plasma glucose, **D)** plasma insulin, **E)** serum testosterone, **F)** serum erythropoietin, **G)** serum GDF-15 and **H)** plasma β-CTX. Absolute values and statistical analyses are presented in table 2. n = 10 (individuals) x 4 or 8 (samples).

**Figure S2.** Resting and exercise-related respiratory parameters. Change in **A)** Resting carbohydrate (CHO) oxidation at rest, **B)** CHO oxidation during exercise, **C)** Resting metabolic rate and **D)** Resting metabolic rate (relative to fat free mass). Absolute values and statistical analyses are presented in table 2. Details of exercise variables are presented in table 3. n = 10 (individuals) x 4 or 6 (samples).

**Figure S3.** Fractional synthetic rate (%/day) of proteins annotated into soluble, myofibrillar, and mitochondrial assessed in silico. The box plot represents the interquartile range (IQR; 25th–75th percentile), with the horizontal line indicating the median. Whiskers extend to the minimum and maximum values within 1.5× IQR. FSR (%/day) values for individual subjects are shown as black data points. Gray lines connect values from the same subject across the three periods (FL, EB, ED). The red diamond represents the mean. Pairwise comparisons of the estimated marginal means for the experimental period were conducted using the Bonferroni adjustment for multiple comparisons in the linear mixed-effects model.


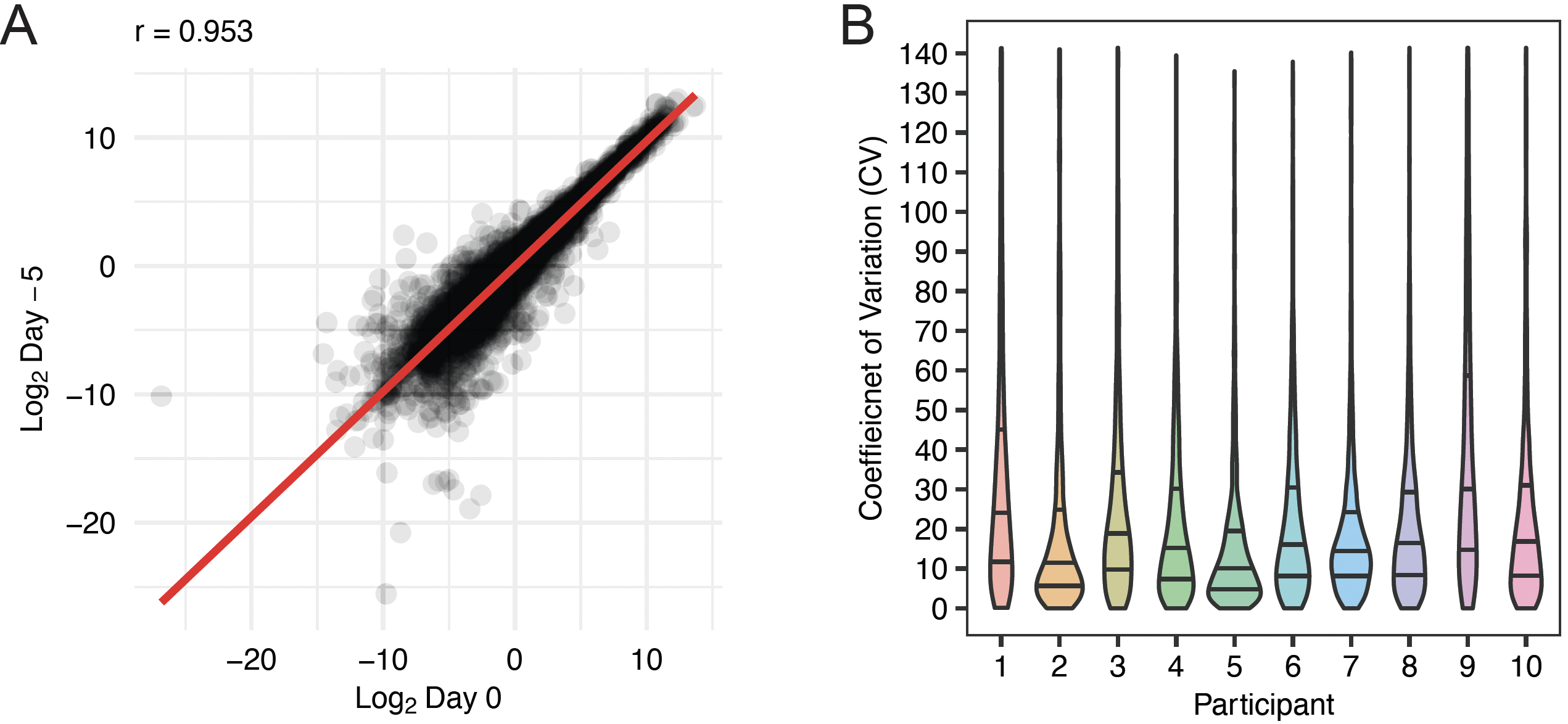


**Figure S4. *Skeletal muscle proteome abundance is stable during the FL condition.*** A, a scatter plot of protein abundance data between Day -5 and day 0 from all 10 participants. B, violin plots of Coefficient of Variation between Day -5 and Day 0 on a protein-by-protein basis.


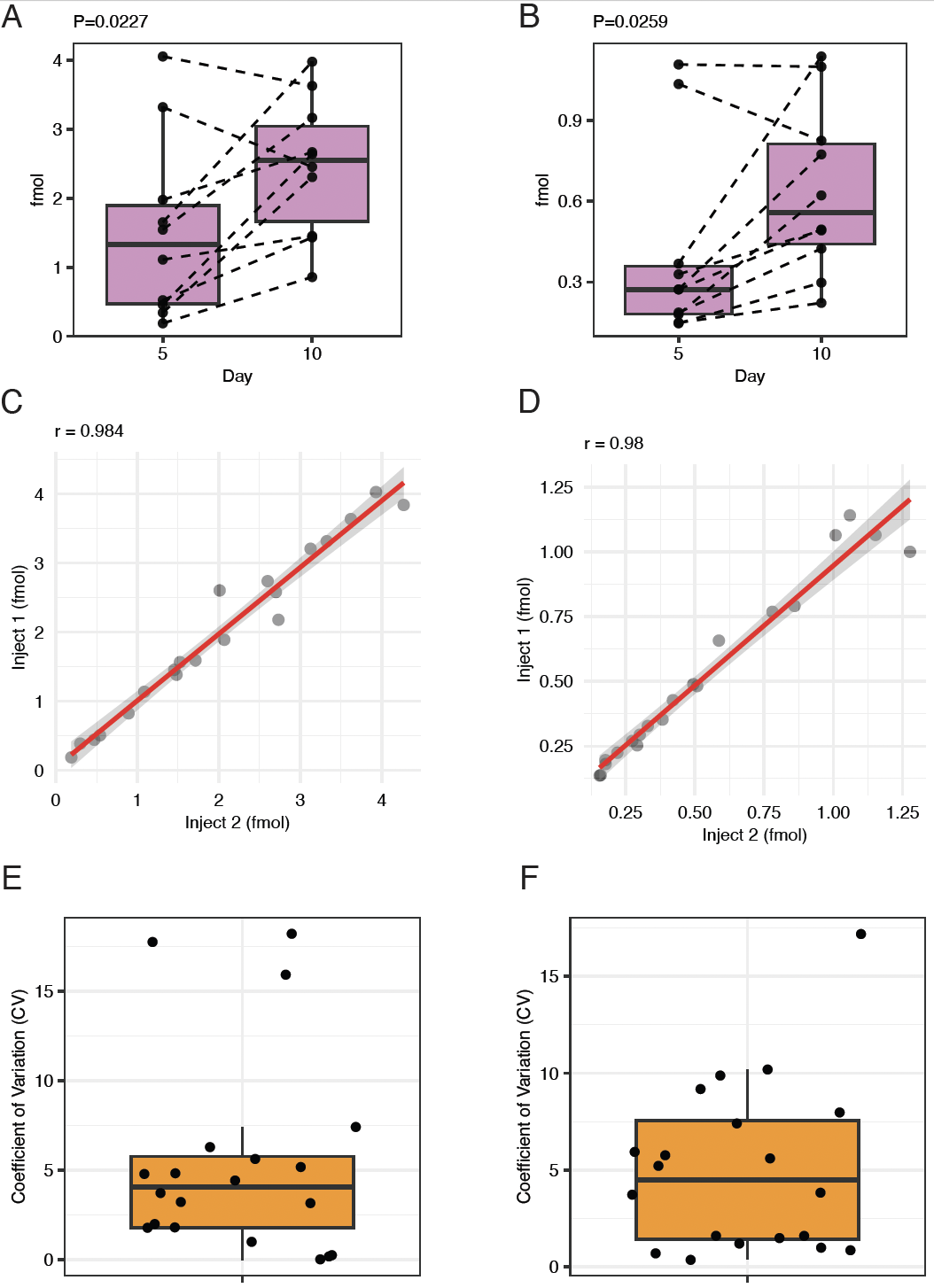


***Figure S5. Targeted proteomic analysis via parallel reaction monitoring verifies PLIN2 and PLIN5 are more abundant in energy deficit.*** Box plot illustrating absolute protein abundance of A, PLIN2 and B, PLIN5. A scatter plot comparing absolute protein abundance between two independent injections into LC-MS in C, PLIN2 and D, PLIN5. Coefficient of Variation (CV) of absolute protein abundance between 2 independent injections into LC-MS in E, PLIN2 and F, PLIN5.

**Figure S6. Box plots of proteins in cluster 1 identified by c-means fuzzy clustering.**

Box plots illustrating changes of protein abundance across the experimental period (38 proteins, all P<0.05). The box plot represents the interquartile range (IQR; 25th–75th percentile), with the horizontal line indicating the median. Whiskers extend to the minimum and maximum values within 1.5× IQR.

**Figure S7. Box plots of proteins in cluster 2 identified by c-means fuzzy clustering.** Box plots illustrating changes of protein abundance across the experimental period (44 proteins, all P<0.05). The box plot represents the interquartile range (IQR; 25th–75th percentile), with the horizontal line indicating the median. Whiskers extend to the minimum and maximum values within 1.5× IQR.

**Figure S8. Box plots of proteins in cluster 3 identified by c-means fuzzy clustering.** Box plots illustrating changes of protein abundance across the experimental period (26 proteins, all P<0.05). The box plot represents the interquartile range (IQR; 25th–75th percentile), with the horizontal line indicating the median. Whiskers extend to the minimum and maximum values within 1.5× IQR.


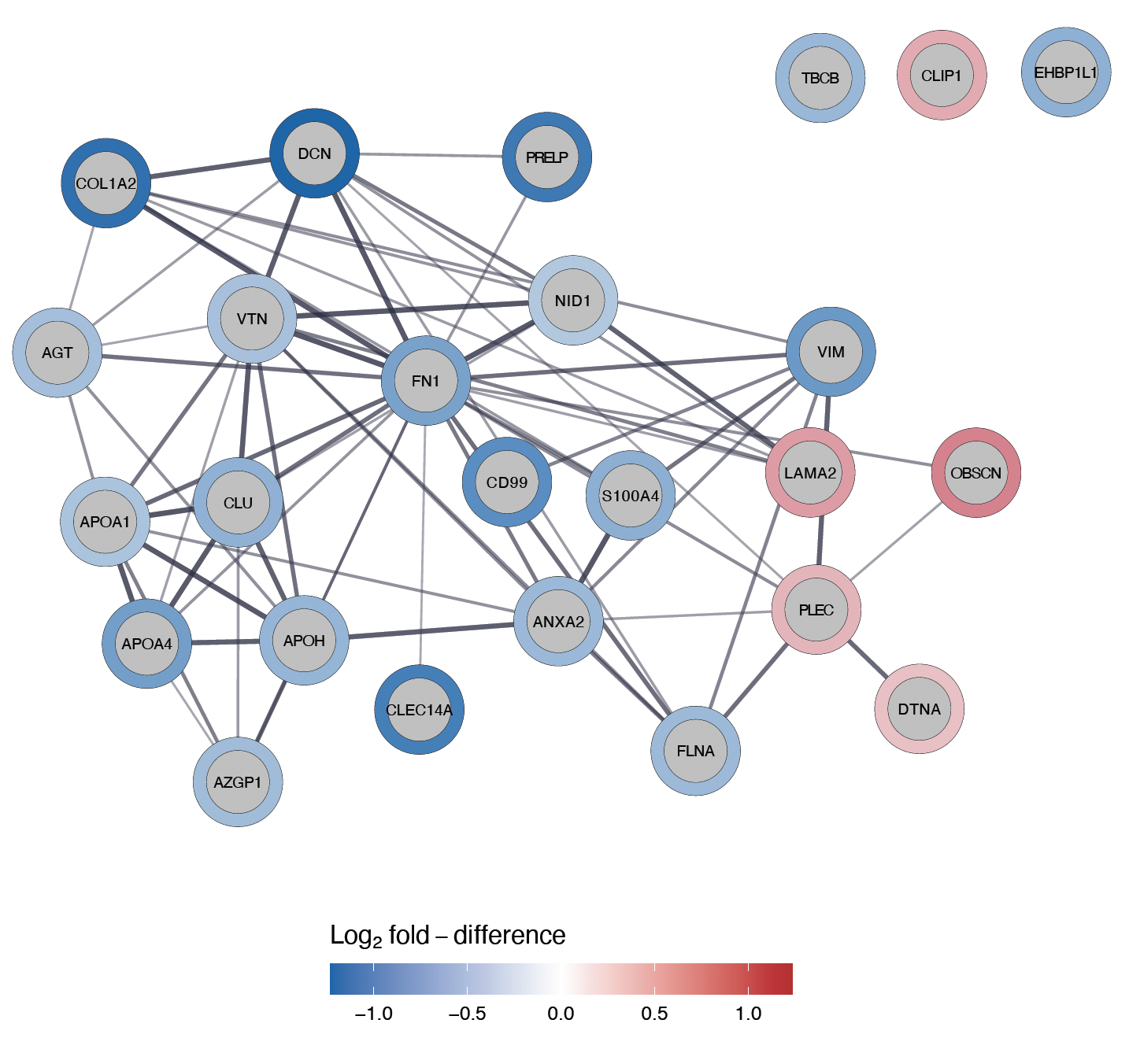


**Figure S9**. STRING network of proteins belonging to the extracellular matrix and cytoskeletal network identified through GO network analyses and independent analysis of significantly different proteins between EB and ED (Supplementary table 3). The boarder colour represents Log_2_ fold-difference in protein abundance between ED and EB.
